# Estimating Total Body Lipid Store of Free‐Ranging Whales In Vivo Using Drone Photogrammetry and Biologging Tags

**DOI:** 10.1002/ece3.72472

**Published:** 2025-11-26

**Authors:** Alec Burslem, Saana Isojunno, Rui Prieto, Mónica A. Silva, Patrick J. O. Miller

**Affiliations:** ^1^ Sea Mammal Research Unit, Scottish Oceans Institute University of St Andrews St Andrews UK; ^2^ Centre for Research Into Environmental and Ecological Modelling University of St Andrews, The Observatory, Buchanan Gardens, University of St Andrews St Andrews UK; ^3^ Okeanos – Institute of Marine Sciences University of the Azores and Institute of Marine Research – IMAR Horta Portugal

## Abstract

Lipid reserve mass is a key indicator of individual fitness and population health in wild animal populations, but no method currently exists to measure it in large aquatic species for which capture is impractical. Here, we develop and employ a new method for quantifying lipid stores in a free‐ranging cetacean, the sperm whale ( *Physeter macrocephalus* ). This method combines UAV (drone) photogrammetry‐based estimates of body volume with tag‐derived tissue density to estimate lipid store mass to within an estimated 18% on average. Statistical analysis cross validating volume and density‐based condition indicators confirmed the predicted relationships between body volume, tissue density and body composition. On average, for a whale 12 m in length, an increment in volume of 1 m^3^ was predicted to be accompanied by a 6.3% increase in total body lipid content, while a decrease of 1 kg m^−3^ in tissue density was predicted to reflect a 5.1% increase in total body lipid content. These results support the effectiveness and encourage continued use of densitometric and morphometric methods for quantifying body condition in cetaceans and highlight the value of simultaneously employing both approaches to directly estimate lipid reserve mass. We anticipate that this method could be extended to other aquatic taxa allowing, for example, major life history events or exposure to stressors to be tied to absolute measures of lipid energy store.

## Graphical abstract

Lipid reserve mass is a key indicator of individual fitness and population health in wild animal populations, but no method currently exists to measure it in large aquatic species for which capture is impractical. Here, we develop and employ a new method for quantifying lipid stores in a free‐ranging cetacean, the sperm whale (Physeter macrocephalus). This method combines UAV (drone) photogrammetry‐based estimates of body volume with tag‐based estimates of tissue density to estimate lipid store mass to within an estimated 18% on average. We anticipate that this method could be extended to other aquatic taxa allowing, for example, major life history events or exposure to stressors to be tied to absolute measures of lipid energy store.

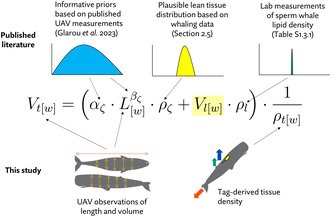

## 1 Introduction

Lipid‐store body condition, the size of an individual's available lipid store as a proportion of total body mass or volume, is an important variable in the study of animal behaviour, physiology and conservation biology because it reflects the size of the animal's energy resources relative to metabolic demand. Individuals in better condition exhibit improved fitness through higher fecundity and survival probabilities (Hall et al. 2001; Stewart et al. 2021). In animals wherein vital rates are difficult to study directly (due to e.g., high mobility, slow life histories etc.), such as marine mammals, body condition can be a key proxy for assessing the health and fitness of individuals and, ultimately, populations (Booth et al. 2020; Pirotta et al. 2018). Body condition also represents a key internal state variable against which animals balance their behavioural decision‐making. Such decision‐making includes timing and investment in energetically costly life history events such as migration and lactation, as well as how animals balance the risk of starvation against response to biological threats and anthropogenic stimuli (Moran et al. 2020). Lipid‐store body condition may therefore be important for contextualising the drivers of behavioural threat responses and understanding their consequences (Bejder et al. 2009; Burslem et al. 2022; Siegal et al. 2022).

Unfortunately, body condition is challenging to measure in large, free‐ranging aquatic taxa such as large whales. Direct measurement of total body lipids requires lethal sampling, which has historically been achieved through whaling and is now generally considered ethically unacceptable in these taxa. Lethal sampling also prohibits longitudinal study, which is important for understanding the mechanistic drivers and consequences of body condition. Non‐lethal alternatives commonly used with terrestrial mammals, polar bears ( *Ursus maritimus* ) and pinnipeds, such as isotopic dilution and bioelectrical impedance (Speakman 2001), require temporary capture, which is impractical for large cetaceans. Measuring the lipid content of blubber samples obtained by remote biopsy is relatively tractable, but whole‐body lipid stores are often not well represented by this method (Christiansen et al. 2020; Ryan and Kershaw 2022). In some marine mammal species, significant lipid stores may be located within the body cavity and thus be unavailable to remote biopsy. Improved metrics of body condition have recently been identified as a high‐priority research area for informing the study of marine mammal energetics (McHuron et al. 2022).

There are currently two leading indirect methods of characterising body condition in free‐ranging marine mammals: overhead photogrammetry and tissue density derived from animal‐attached tag data (Castrillon and Bengtson Nash 2020). Overhead photogrammetry uses imagery obtained from an aircraft or unmanned aerial vehicles (UAVs) to quantify changes in girth or total animal volume relative to length (Castrillon and Bengtson Nash 2020; Christiansen et al. 2016). By contrast, tissue density estimation uses hydrodynamic performance modelling of biologging data to calculate tissue density, which is driven by the proportion of low‐density fats relative to higher density muscle, bone, blood and other lean material (Castrillon and Bengtson Nash 2020; Miller et al. 2004). These morphometric and densitometric methods show broad agreement as indices of body condition in, for example, humpback whales ( *Megaptera novaeangliae* , Aoki et al. 2021) but have not yet been cross‐validated in deep‐diving odontocetes. Photogrammetry can measure the volume and morphology of the body with high accuracy, but not the makeup of the underlying tissue (i.e., lean vs. adipose). It is therefore incapable of detecting differences in body condition attributable to altered body composition. Conversely, tissue density contains information about total body lipid percentage, independent of where lipid stores are located within the body but does not measure the absolute size of the body, or any compartments within it. To know the size of the lipid (and therefore energy) store in real units, it is necessary to know both the size and lipid percentage of the animal's body. Biological interpretations of results from either method may therefore benefit from improved knowledge on how each corresponds to the underlying lipid‐store body condition state.

Here, we demonstrate a novel method to use simultaneous UAV‐based morphometry and tag‐based tissue densitometry in a complementary fashion to quantify body volume and composition in free‐ranging sperm whales. We (1) deployed biologging tags and conducted UAV overflights on free‐ranging sperm whales (2) quantified whale volume and density using UAV photogrammetry and hydrodynamic modelling of tag data, respectively (3) developed a formal model of the volume, composition and density of sperm whale tissues (4) fitted a Bayesian estimation framework to test for a shared underlying body condition state driving variability in morphometric and densitometric measures and (5) incorporated lean and lipid density information from historical whaling records to convert volume and density observations to absolute total‐body lipid store estimates.

## 2 Materials and Methods

### 2.1 Field Data Collection

Fieldwork was conducted off the Azores archipelago (nursery, foraging and breeding grounds for all sex and age classes) during June–September 2021 and 2022, and separately off Northern Norway (foraging grounds for sexually segregated males) in June–September 2009–2019 as part of the 3S active sonar trials (Kvadsheim et al. 2021; Lam et al. 2018; Miller et al. 2011).

#### 2.1.1 Tags and Tagging

High resolution movement data were collected using DTag version 2 or 3 (Johnson and Tyack 2003), either alone or as part of the ‘mixed Dtag’ modular sensor package (Kvadsheim et al. 2021). All tags recorded high resolution (50–250 Hz) depth, 3‐axis magnetism, and acceleration, were attached non‐invasively using suction cups and were applied from small boats using either a 12 m cantilevered pole or a 7 m hand‐pole. See Appendix S1.1 for additional details.

#### 2.1.2 UAV Photogrammetry

A DJI Phantom 4 Pro UAV was used for all flights performed in the Azores in 2021–2, while a DJI Phantom 4 was used for flights in Norway. Both platforms carried a custom‐built laser altimeter and datalogger system to measure height above the whale. Overhead photogrammetry videos of sperm whales were obtained by flying the UAV above the animals at an elevation of 13–23 m in winds not exceeding 6 m s^−1^ and sea state not exceeding Beaufort 3. The camera was operated using DJI Pilot software (DJI 2020). All images of a given whale were taken within the same day. Suitable image frames were isolated and matched to height data streams using a custom‐written MATLAB script and unless otherwise stated (see Appendix S1.1) only images with a valid altimeter reading within 1 s and meeting established quality criteria were used in downstream analyses. Both systems were checked and, where necessary, adjusted for systematic measurement bias. See Appendix S1.1 for additional details.

#### 2.1.3 Research Ethical Statement

Tagging and drone flights were undertaken under research permits LMAS‐DRAM/2020/06 and 2021/12, issued by the Azores Regional Directorate for Sea Affairs (Direção Regional dos Assuntos do Mar; DRAM) and held by coauthor MAS. All responses to UAV overflights and tagging approaches were scored on a scale of 0–3 according to standard behavioural response criteria (Weinrich et al. 1992). The severity of responses to both tagging and UAV overflights was recorded (Table S1) and if a response of type 3 (severe and prolonged) was observed, research activities with that individual were immediately ceased. Research activities were approved by the Animal Welfare and Ethics Committee at the University of St Andrews.

### 2.2 Data Processing

#### 2.2.1 Tissue Density

Raw tag data were downsampled to 5 Hz and converted to animal depth, orientation and fluke stroking time series using established methods (Johnson and Tyack 2003; Martín López et al. 2016). Average density of all tissues was estimated from movement data during gliding periods (i.e., with no active stroking, standardised by subdividing into 5 s ‘subglides’ for analysis) using the methods detailed in Miller et al. (2016). If the same animal was tagged more than once, only one tag record was analyzed.

#### 2.2.2 Estimation of Total Body Volume

Whale total length (L) and width at 5% intervals along the body were measured using the Morphometrix software package (Torres and Bierlich 2020). Volumes were estimated from these measurements following the methods described by Christiansen et al. (2019) and using the body site‐specific height: width ratios estimated for sperm whales from a combined sample of adults and calves by Glarou et al. (2023). The volume of each segment (V_[__s]_; the region between adjacent measurement sites) was modelled as a series of infinitesimal ellipses (Christiansen et al. 2019; Equation 1 therein). Segment volumes were then summed to give the estimated total body volume (Christiansen et al. 2019; Equation 2 therein).

### 2.3 Development of the Lipid Store Model

#### 2.3.1 Overview

**FIGURE 1:**
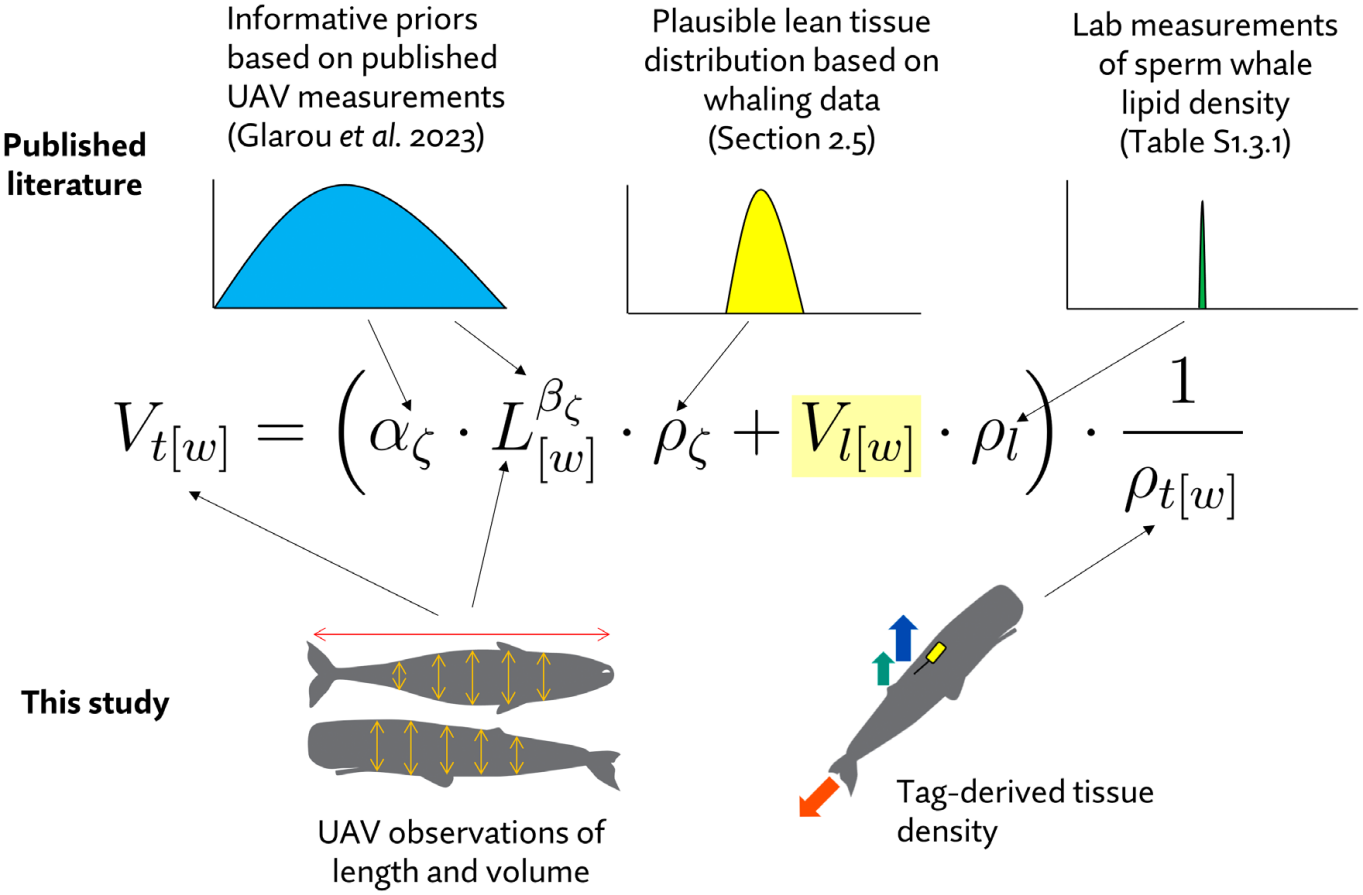
Conceptual illustration of the information synthesized to estimate total body lipid volume (highlighted term). Equation shows the functional form linking the length, density and volume of a whale body consisting of lean (ζ) and lipid store (l) tissue compartments. See Table 1 for full parameter descriptions. Boxes and arrows are used to illustrate how terms in the model were informed in the present analysis. See Sections 2.3 ‘Overview’ and 2.4 ‘Model specification’ for details.

Our method exploits the physical relationships between the volume, density and composition of a body to estimate the biologically relevant, but unobserved, driver of body volume and density i.e., lipid‐store state. Mathematical notation is summarised in Table 1. The modelling approach and sources of information synthesised therein are illustrated conceptually in Figure 1.

**TABLE 1:** Mathematical notation.

Notation	Description	Units
*Parameters*		
ρ	Density	kg m^−3^
M	Mass	kg
P	Proportion	—
V	Volume	m^3^
L	Length	m
H	Height	m
W	Width	m
*Subscripts*		
l	Lipid	
p	Protein	
a	Mineral ash	
w	Water	
g	Gas	
t	Tissue	
*ζ*	Lean tissue compartment	
τ	Reserve compartment, indicates the tissue compartment which varies independently of length and can be considered to indicate body condition	
γ	Reference body the volume and mass of which vary as a function of length alone and can be considered to indicate standard, lean, or structural tissue	
b	Whole body (tissue and gas spaces)	
u	‘True’ hidden value	
*Indices*		
$\left[w\right]$	Individual whale index	
$\left[c\right]$	Compartment index	
$\left[i\right]$	Image index	
$\left[s\right]$	Segment index	
*Modifiers*		
$\bar{x}$	Population average of *x*	
δx	Difference in *x*	

*Note:* Includes additional notation needed to interpret full derivations and likelihoods given in Appendices S1.2 and S1.3, respectively.

The relationship between volume (V) and density (ρ) of an n‐compartment body, each (e.g., tissue) compartment with its own separate density, can be stated as:

(1) $$\rho =\frac{\sum_{c=1}^{n}\rho_{c}\cdot V_{c}}{V}$$

Given the proportional volume and mass of all compartments must sum to one, then if the overall density of the whole body, the density and proportional masses of n‐1 compartments (either separately or combined), and the density of the final compartment ($\rho_{n}$) are known, it is possible to solve for the proportional mass of the final compartment analytically (see Appendix S1.2 for the full derivation):

(2) $$P_{n}=\rho_{n}\cdot \left(\frac{1}{\rho }-\sum_{c=1}^{n-1}\frac{P_{c}}{\rho_{c}}\right)$$

Further, if the volume of the whole body is also known, then it is possible to compute the absolute mass and volume of that compartment. Simultaneous observations of whale body volume and density in this dataset allow for the exploitation of these equalities to estimate the lipid store of free‐ranging sperm whales, given specifications of a suitable reference body (non‐lipid part of the body as in Figure 1). Since reference bodies must be based on direct quantification of tissue‐specific lipid stores, which can usually only be quantified post‐mortem, we constructed distributions of estimates using Monte Carlo simulations based on historical whaling data, with predictions validated against independent datasets (see Section 2.5 Monte Carlo simulations of reference body parameters).

#### 2.3.2 Densitometry

To solve for the proportion of an animal's body that consists of lipid (hereafter, lipid proportion, $P_{l}$), given an observed tissue density ($\rho_{t}$) and a known lipid density ($\rho_{l}$), it is necessary to specify a reference body (γ) to which some proportional energy storage component (τ), is assumed to have been added (or removed).

The equation to calculate the total body lipid proportion from a given tissue density ($\rho_{t}$) and reference body *γ* takes the following general form (Siri 1956; Equation 7 therein):

(3) $$P_{l}=\frac{\rho_{\tau }\cdot \rho_{\gamma }}{\rho_{t}}\cdot \left(\frac{P_{\mathrm{l\tau}}-P_{\mathrm{l\gamma}}}{\rho_{\gamma }-\rho_{\tau }}\right)-\frac{\rho_{\tau }\cdot P_{\mathrm{l\tau}}-\rho_{\gamma }\cdot P_{\mathrm{l\gamma}}}{\rho_{\gamma }-\rho_{l}}$$

Without specifying the lipid proportion of the reference body $P_{\mathrm{l\gamma}}$, we can still solve for the difference in lipid proportion ($\delta P_{l}$) between the body and the reference body ($\delta P_{l}=P_{l\left[w\right]}-P_{\mathrm{l\gamma}}$):

(4) $$\delta P_{l}=\frac{1}{\rho_{t}}\cdot \left(\frac{\rho_{\gamma }\cdot \rho_{\tau }}{\rho_{\gamma }-\rho_{\tau }}\right)-\frac{\rho_{\tau }}{\rho_{\gamma }-\rho_{\tau }}$$

(Siri 1956; Equation 6 therein). Therefore, specifying the reference body to only be comprised of lean tissues with a known density $\rho_{\zeta }$, we can express the lipid proportion by setting $\rho_{\gamma }=\rho_{\zeta }$ and, again equating energy and lipid stores ($P_{\mathrm{l\tau}}=1$, $\rho_{\tau }=\rho_{l}$):

(5) $$P_{l}=\delta P_{l}=\frac{1}{\rho_{t}}\cdot \left(\frac{\rho_{\zeta }\cdot \rho_{l}}{\rho_{\zeta }-\rho_{l}}\right)-\frac{\rho_{l}}{\rho_{\zeta }-\rho_{l}}$$

In comparison with the general form Equation (3), the lean reference body Equation (5) offers a simpler form and assumes only one unmeasured constant: lean density ($\rho_{\zeta }$), which is expected to be less variable among whales and populations than expected lipid proportion ($P_{l}$).

### 2.4 Statistical Models of Body Length, Volume and Density

#### 2.4.1 Setting the Hypothesis

Morphometric body condition may be conceptualised as the ‘fatness’ of an animal, represented mathematically as the residual volume of an animal given its length. Therefore, specifying tissue density as a predictor of volume, in addition to length, represents the basis for testing the hypothesis that animals which appear fatter morphologically also have greater percentage lipid (and lower tissue density). We therefore predicted a strong correlation between morphometric and tissue density‐based indicators of lipid‐store body condition.

#### 2.4.2 Model Specification

Statistical models were fitted in a Bayesian framework to allow for the inclusion of informative priors representing a priori information from the published literature and parameter imputation for animals which were only partially observed. For example, for whales where UAV, but not tag data (and therefore density and composition information) are available, posterior distributions for body composition could be imputed from the fitted model. Similarly, measurement error was estimated using information pooled from the full dataset and was propagated within the model to all whale length and volume estimates, including those where only one image was available and for which individual error estimates could therefore not be calculated directly. The model specification was developed and tested using realistic simulated data to confirm that the parameters used to simulate the data were identifiable and accurately recovered (See Appendix S1.3 for details).

The model was specified with a hidden process linking underlying body length, volume and tissue density and an observation process for the measured tissue density, body length, and body volume. Individual differences in lipid percentage from the population average were estimated within the model. The process model was then specified to estimate slopes that related individual volume to lipid percentage (*β*_*δPl*_) and length (*β*_*v*_). This process model thus tested the hypothesis that individuals with greater residual volumes also had a greater lipid percentage.

Measurement error was specified at the level of individual whales, allowing more certain observations of length, volume or tissue density to exert greater influence on the linear predictors than less certain ones. Tissue density measurement error was specified based on the posterior output by the upstream hydrodynamic model (Aoki et al. 2021). Stochastic measurement error (i.e., precision, as opposed to the systematic measurement inaccuracy dealt with as described in Appendix S1.1) associated with UAV measurements of whale length and volume observations, was estimated within the model using repeated observations of the same whale taken within the same day. Previous similar analyses have instead specified measurement precision parameters based on measurements of objects of known length, often in a controlled environment such as a harbour, which are then generalised to the field measurements of individual whales (e.g., Bierlich et al. 2021). This approach introduces the implicit assumption that the resulting precision estimate is representative of that in the true measurement context across observation events (i.e., different whales and days). While this assumption is likely to be necessary and reasonable in many circumstances, our photogrammetry data were collected across a variety of conditions capable of impacting altimeter error (e.g., Beaufort Sea state range: 0–3) and expected to vary across observation events. They may also contain additional imprecision resulting from other processes such as changes in whale pose not detected or excluded by the image scoring protocol. We therefore opted instead to adopt a repeated‐measures approach. This allows volume and length measurement error (and therefore influence on the model) to vary across whales and days, and has the additional advantage of estimating total imprecision using the observed measurements resulting from the full, context‐specific data (and therefore error) generation process (i.e., measurements of a given whale in a given set of measurement conditions) as is best practice (Drosg 2007; White 2008).

Informative priors (Table 2) encoding information from the published literature were specified for global average tissue density ($\bar{\rho_{t}}$) and allometric volume parameters, i.e., the log‐scale slope (*β*_*v*_) and intercept (*α*_*v*_) describing the relationship between length and expected volume. The parameter *β*_*δPl*_ was specified a weakly informative prior, intended to promote a conservative inference by avoiding spurious inference of a large effect (Lemoine 2019). All other parameters were specified uniform priors which were uninformative except to restrict the sampler to between plausible minima and maxima.

**TABLE 2:** Informative priors specified in the Bayesian analysis.

Parameter	Description	Prior	Source(s)
$\log\left(\alpha_{v}\right)$	Allometric log intercept	$∼\text{Gaussian}\;\left(-4.048,0.08646\right)$	(Glarou et al. 2023 & pers. comm.)
$\log\left(\beta_{v}\right)$	Allometric scaling exponent	$∼\text{Gaussian}\;\left(2.861,0.04094\right)$	(Glarou et al. 2023 & pers. comm.)
$\bar{\rho_{t}}$	Population mean tissue density	$∼\text{Gamma}\;\left(5412,5.255\right)$	(Miller et al. 2004)
$\beta_{\mathrm{\delta Pl}}$	Linear predictor of volume as a function of density.	$∼\text{Gaussian}\;\left(0,50\right)$	

*Note:* The distribution parameters are mean and standard deviation, and shape and rate for Gaussian, and Gamma distributions, respectively. The informative prior for β_δPl_ does not represent a prior state of knowledge, but rather serves as a conservative prior intended to promote a parsimonious model. All other parameters were specified uniform priors which were uninformative except to restrict the sampler to values betweeen the minimum and maximum ones considered plausible (also see model specification code and additional technical details in Appendix S1.3).

#### 2.4.3 Model Fitting

Models were fitted in the R programming environment using the NIMBLE MCMC construction and sampling engine (de Valpine et al. 2017). Each model was sampled in three chains with different initial values. Sampling consisted of 100,000 total iterations, with the first half discarded as burn‐in and remaining posterior samples down‐sampled by a factor of 50. Model convergence was assessed using the Gelman‐Rubin statistic and visual inspection of the Markov chains and posterior densities. Plots of posterior samples were inspected for collinearity to confirm that estimates were well identified. Posterior predictive checks were used to assess model fit and that the model had adequately captured the observation variability present in the dataset.

### 2.5 Monte‐Carlo Simulations of Reference Body Parameters

Reference body densities and lipid proportions were generated to convert tissue differences in lipid proportion $\delta P_{l\left[w\right]}$ to absolute lipid proportion $P_{l\left[w\right]}$. reference body values were specified as Monte Carlo draws rather than point values to allow parameter uncertainty to propagate through to final lipid store estimates. Samples for reference body composition and density were generated using Monte Carlo simulations based on raw and summary data from the published literature (Clarke 1978a, 1978b; Evans et al. 2003; Lockyer 1991; Lockyer 1976; Ohno and Fujino 1952; Omura 1950; Watanabe and Suzuki 1950).

Mammalian bodies consist of different tissue compartments, such as adipose tissue and muscle, which each contribute various proportions to total body mass. Further, each tissue compartment can itself be considered a compartmentalised mixture of mutually insoluble components: water (w), lipids (l), proteins (*p*) and mineral ash (a). Therefore, the lean and total mass, volume and density of a sperm whale body can be approximated as described in Equations ((6), (7), (8), (9)). Terms in grey are included to calculate total body tissue mass, volume or density, but left out for lean equivalents ($M_{\zeta }$, $V_{\zeta }$ and ρ_ζ_, respectively):

(6) $$\begin{array}{c} M_{\zeta \left[w\right]}=\sum_{c=1}^{8}M_{\left[w,c\right]}\cdot P_{p\left[w,c\right]}+M_{\left[w,c\right]}\cdot P_{a\left[w,c\right]}\\ \;+M_{\left[w,c\right]}\cdot P_{w\left[w,c\right]}+M_{\left[w,c\right]}\cdot P_{l\left[w,c\right]} \end{array}$$

(7) $$\begin{array}{c} V_{\zeta \left[w\right]}=\sum_{c=1}^{8}\frac{M_{\left[w,c\right]}\cdot P_{p\left[w,c\right]}}{\rho_{p}}+\frac{M_{\left[w,c\right]}\cdot P_{a\left[w,c\right]}}{\rho_{a}}\\ \;+\frac{M_{\left[w,c\right]}\cdot P_{w\left[w,c\right]}}{\rho_{w}}+\frac{M_{\left[w,c\right]}\cdot P_{l\left[w,c\right]}}{\rho_{l}} \end{array}$$

(8) $$\rho_{\zeta \left[w\right]}=\frac{M_{\zeta \left[w\right]}}{V_{\zeta \left[w\right]}}$$

where

(9) $$M_{\left[w,c\right]}=f\left(L_{w}\right)$$

and *c* is the set of tissue compartments, which sum to 1 by proportion mass: *c* = {*Skeleton, Muscle, Organs, Viscera, Blubber, Sound production apparatus, Other, Blood*}$c=\left\{\text{Skeleton},\text{Muscle},\text{Organs},\text{Viscera},\text{Blubber},\text{Sound production apparatus},\text{Other},\text{Blood}\right\}$.

The above calculations were performed for 10^6^ simulated sperm whales with body lengths of 8–17 m using empirically derived allometric and compositional models. The specific parameters and corresponding sources used for sperm whales are described and illustrated in the Tables S2 and S3; Figure S1. Both lean and total masses, volumes, compositions and densities were calculated, to represent ‘lean’ and ‘standard whale’ reference bodies. Neither empirical data (individual total length ~ tissue density Spearman's *ρ* = 0.43) nor our simulation results indicated a systematic relationship between body size (which is also the best available indicator of sex) and body composition across the relevant range of sizes, so both reference body distributions were specified to be univariate.

#### 2.5.1 External Validation

Simulations were run with all tissue constituents, including lipids, and the results compared with estimates from the published literature. The simulated total body tissue densities were compared with those reported by Miller et al. (2004) and simulated lipid stores were compared with whole body yields from historical whaling operations (Irvine et al. 2017).

### 2.6 Post Hoc Calculations

#### 2.6.1 Total Body Lipid Stores

Since the densitometric approach uses estimated lipid store as a proportion by mass, it is necessary to calculate body mass to obtain absolute total body lipid store. We converted tissue density to total body density ($\rho_{b}$), including the gas component, as follows:

(10) $$V_{g}=P_{g}\cdot 10^{-6}\cdot \rho_{t}$$

(11) $$M_{g}=V_{g}\cdot \rho_{g}$$

(12) $$\rho_{b}=\frac{\rho_{t}+M_{g}}{1+V_{g}}$$

where $\rho_{g}$ is the density of air at one atmosphere: 1.225 kg m^−3^ and multiplication by 10^−6^ converts units from ml to m^3^. Total body mass $\left(M_{b}\right)$ is then:

(13) $$M_{b}=V_{b}\cdot \rho_{b}$$

Absolute lipid proportion $P_{l\left[w\right]}$ was computed using Equation (5) across all posterior $\rho_{t\left[w\right]}$ samples (*n* = 3000) with corresponding lean density values drawn from the outputs of the Monte‐Carlo simulations. Finally, lipid proportion was multiplied by $M_{b}$ to estimate total body lipid mass ($M_{l}$).

## 3 Results

The fieldwork in Norway and the Azores resulted in 47 independent tag deployments suitable for tissue density analysis, of which 16 also had UAV overflights. These were combined with 4 UAV overflights of untagged animals (total *n* = 51 whales, 47 tag deployments).

### 3.1 Body Size and Condition in Sperm Whales

**FIGURE 2:**
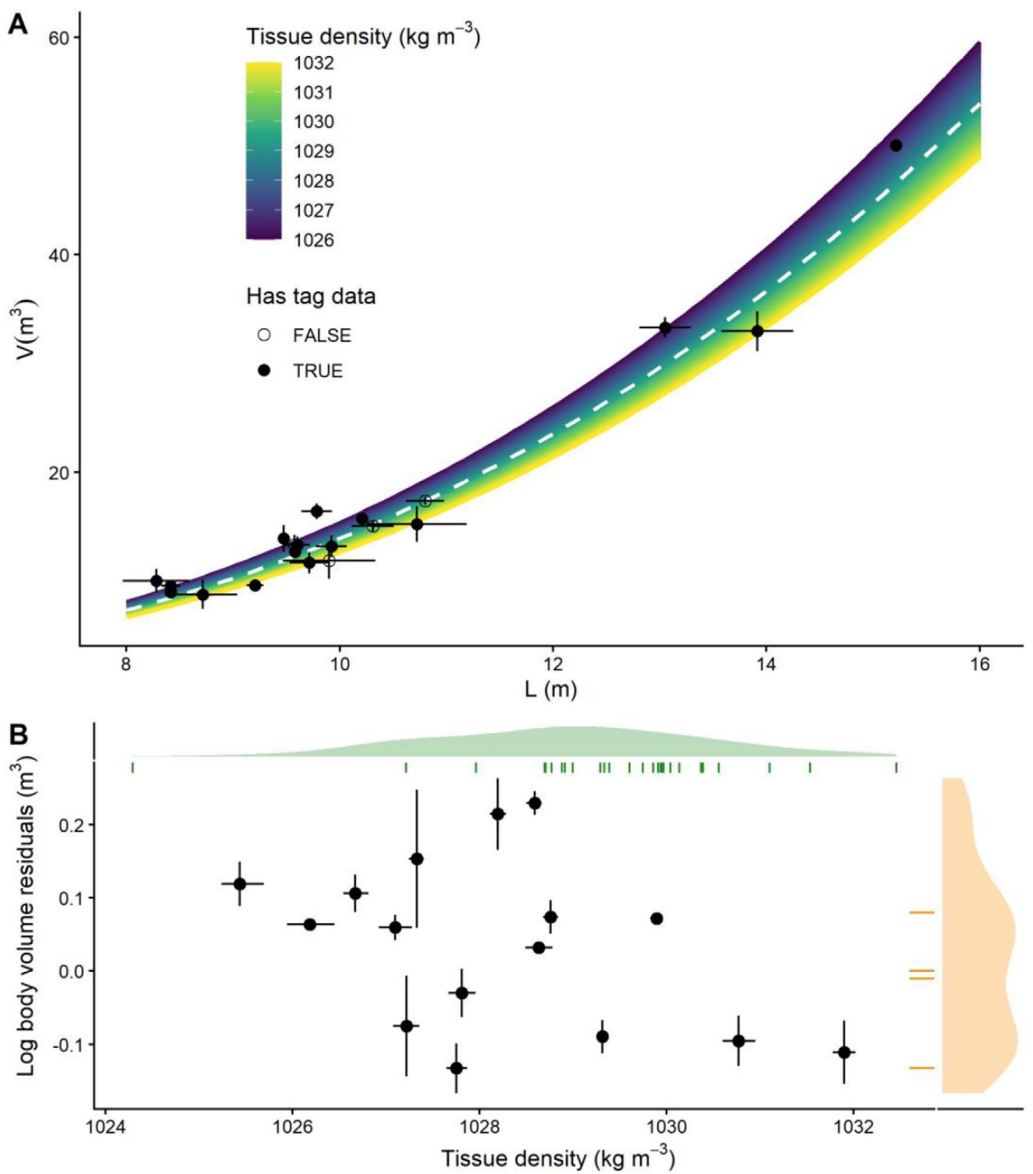
Measured body volumes and their relationship with length and tissue density. (A) Raw length and volume estimates based on photogrammetry points = mean ±95% confidence intervals and predictions of volume shows as a function of length alone (dashed white line) and length plus tissue density (coloured prediction surface) based on posterior mean coefficients. Since UAV photogrammetry supplies both length and volume data this plot shows animals for which only UAV data are available (open points, *n* = 4) and those for which tag derived‐tissue density are also available (filled points, *n* = 16) (B) Plot of tissue density and residuals of allometric (length alone) model of total body volume i.e., volumetric body condition. Rug plots show residual volume and tissue density for whales where only one variable was available. For whales where both variables are available the scatter plot shows the relationship between them: Mean ±95% credible intervals (density) and confidence intervals (volume). Axis density plots show the probability density for each variable across the full dataset.

Individual body density ranged from 1024.3 to 1032.5 and the global mean was estimated at 1029.7 ± 0.47 kg m^−3^ (mean ±95% CRI). Individual average photogrammetric measurements of body length and volume ranged from 8.3 to 15.2 m (mean = 10.2, three large putative males [≥ 13.05 m], 17 unknown sex [≤ 10.8 m]) and 8.8–50.06 m^3^ (mean = 16.74), respectively (Figure 2A and Table 3). The posterior mean allometric coefficients estimated in the hypothesis testing model corresponded to a response scale length‐to‐volume function of $V=0.018\cdot L^{2.88}$ (Figure 2A and Table S4).

**TABLE 3:** Summary data for the whales imaged and/or tagged for this study.

Whale ID	Location	No of 5 s sub‐glides.	Tissue density (kg m^−3^ ± CRI)	No. of images	Length (m ± CI)	Volume (m^3^ ± CI)	Difference in lipid composition from average (% ± CRI)	Lipid mass (metric tonnes ±CRI)	Of which is spermaceti (approx. metric tonnes)
sw19_250ab	Norway	118	1029.9 ± 0.07	1	15.21	50.06	−0.4 ± 0.46	10.1 ± 2.13	2.29
sw19_253abc	Norway	85	1029.32 ± 0.07	6	13.91 ± 0.83	32.97 ± 4.48	−0.12 ± 0.45	7.55 ± 0.94	1.63
sw21_196a	Azores	79	1027.1 ± 0.18	3	8.41 ± 0.07	8.97 ± 0.45	0.94 ± 0.48	2.06 ± 0.22	0.36
sw21_211a	Azores	41	1030.77 ± 0.17	5	9.71 ± 0.41	11.67 ± 2.19	−0.81 ± 0.48	2.57 ± 0.34	0.55
sw21_215a	Azores	80	1027.81 ± 0.15	2	9.92 ± 0.21	13.2 ± 1.41	0.59 ± 0.45	3.12 ± 0.49	0.58
sw21_221a	Azores	56	1028.59 ± 0.08	4	9.78 ± 0.28	16.43 ± 1.45	0.23 ± 0.46	3.29 ± 0.85	0.64
sw21_230a	Azores	126	1027.33 ± 0.08	2	9.48 ± 0.03	13.9 ± 1.74	0.83 ± 0.45	2.98 ± 0.53	0.54
sw21_230b	Azores	58	1027.21 ± 0.11	0	—	—	0.88 ± 0.45	—	—
sw21_232a	Azores	—	—	3	9.57 ± 0.06	13.31 ± 1.57	0.54 ± 2.37	2.91 ± 0.63	0.54
sw21_232b	Azores	38	1024.29 ± 0.1	0	—	—	2.29 ± 0.45	—	—
sw21_242a	Azores	34	1025.44 ± 0.23	2	8.42 ± 0.13	9.53 ± 0.82	1.73 ± 0.5	2.24 ± 0.37	0.36
sw22_181a	Azores	114	1029 ± 0.06	0	—	—	0.03 ± 0.45	—	—
sw22_192a	Azores	37	1031.9 ± 0.12	3	10.72 ± 0.81	15.24 ± 2.81	−1.35 ± 0.46	3.25 ± 0.55	0.75
sw22_201a	Azores	25	1027.22 ± 0.14	4	8.72 ± 0.65	8.75 ± 2.69	0.88 ± 0.46	2.14 ± 0.43	0.38
sw22_201b	Azores	85	1028.2 ± 0.09	3	8.28 ± 0.55	10.03 ± 1.86	0.42 ± 0.46	2.09 ± 0.51	0.38
sw22_209a	Azores	17	—	4	10.31 ± 0.39	15.04 ± 1.15	−0.08 ± 2.21	3.34 ± 0.59	0.67
sw22_210a	Azores	39	1026.67 ± 0.13	3	13.05 ± 0.41	33.3 ± 1.6	1.14 ± 0.45	7.54 ± 0.92	1.42
sw22_216a	Azores	31	1027.76 ± 0.11	3	9.21 ± 0.14	9.6 ± 0.46	0.62 ± 0.46	2.4 ± 0.55	0.44
sw22_223a	Azores	70	1028.76 ± 0.08	4	9.6 ± 0.23	13.33 ± 1.48	0.15 ± 0.46	2.86 ± 0.34	0.56
sw22_224a	Azores	—	—	3	10.8 ± 0.31	17.37 ± 0.61	0.02 ± 2.34	3.86 ± 0.71	0.78
sw22_229a	Azores	26	1028.63 ± 0.15	1	9.58	12.69	0.21 ± 0.46	2.81 ± 0.63	0.54
sw22_234a	Azores	25	1026.19 ± 0.25	1	10.21	15.74	1.37 ± 0.5	3.72 ± 0.85	0.65
sw22_234b	Azores	—	—	2	9.9 ± 0.61	11.86 ± 2.39	−0.44 ± 2.84	2.73 ± 0.83	0.56

*Note:* Tag‐only tissue density data are not reproduced here but are available in the Digital Data Supplement (DS1). Estimated variables are given as mean ±95% CIs (frequentist summary statistics from observed data) or CRIs (credible intervals, Bayesian model estimates). Whales larger than 12.3 m are assumed to be males, but the sex for smaller whales was unknown.

The posterior mean estimate for the linear predictor of body volume from lipid proportion (*β*_*δl*_) was 6.75 (95% CRI range = 2.63–10.96, Table S4), with 99.8% of the posterior samples falling above zero, indicating a positive relationship between proportional lipid store and residual volume (Table S4; Figure 2A,B; Figure S2). Chains were well mixed, parameters were well identified and posterior predictive samples indicated a good fit of the model to the observations (Figures S3 and S4).

### 3.2 Density and Composition of Reference Bodies

**FIGURE 3:**
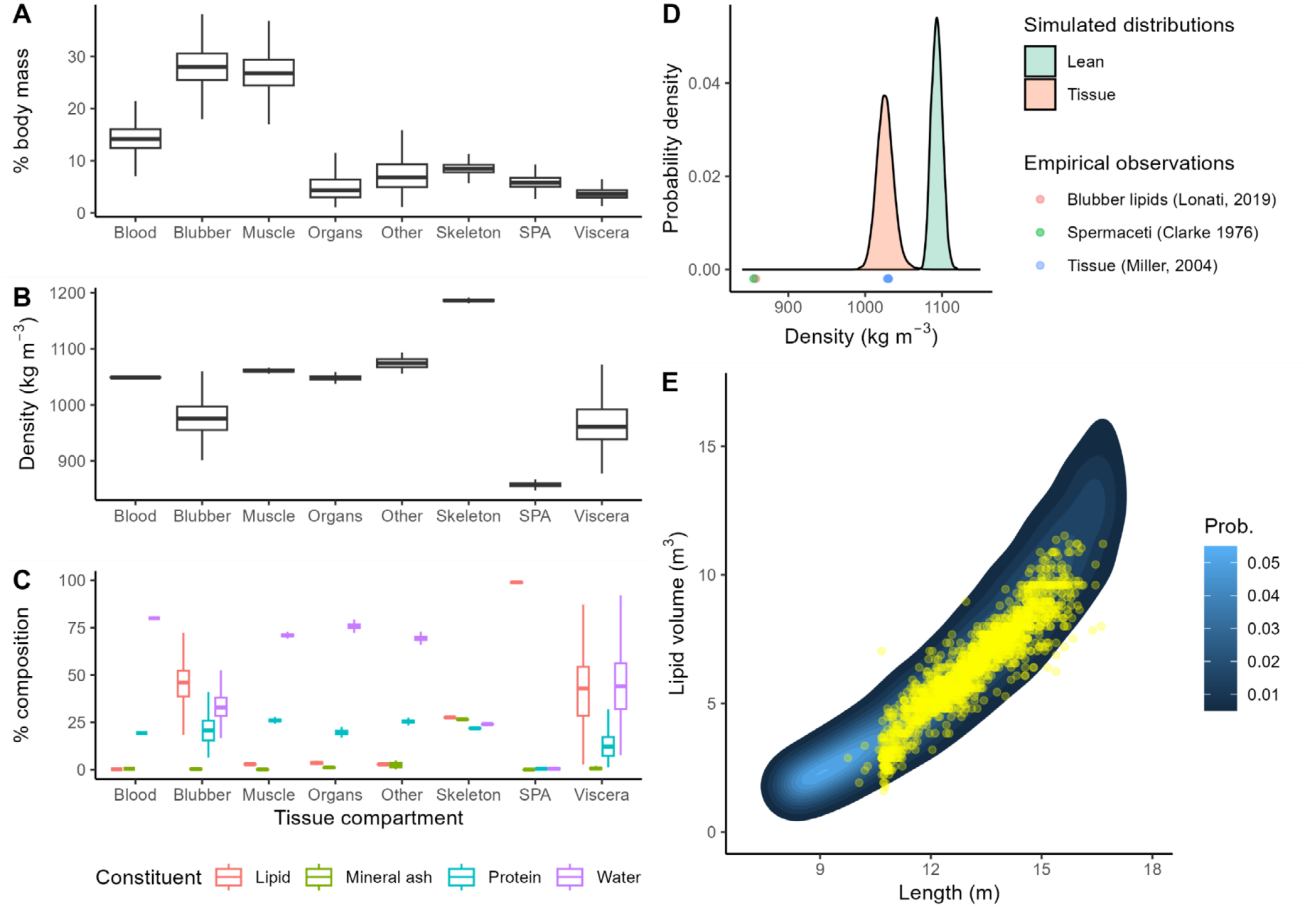
Expected reference body density and composition estimates from Monte Carlo simulations. Top left (A) shows standard boxplots (median ±1st and 3rd quantiles [box] and 1.5× the interquartile range [whiskers]) for each tissue compartment mass as a percentage of total simulated body mass. SPA is sound production apparatus. Middle left (B) shows the same for compartment tissue density. The bottom left (C) shows the % mass composition of each tissue compartment, broken down by four mutually insoluble chemical constituents. Top right (D) shows expected distributions for lean tissue and all tissue. Points show empirical lipid and tissue densities. The bottom right (E) shows the bivariate probability surface for lipid volume (V_l_) and length (L), generated using 10^6^ simulated values, overlaid with published empirical yield values from Irvine et al. (2017). The empirical observations of tissue density and lipid yield in (D, E) are independent from any data used to parameterise the simulations.

Predicted composition, density and contribution to total body mass are shown for individual tissue compartments in Figure 3A–C. The whole body had a predicted physical tissue density of 1026 ± 11.0 kg m^−3^ (mean ± SD) and consisted of 52.8% ± 2.9% water, 20.9% ± 2.1% protein, 23.5% ± 3.7% lipid and 2.6% ± 0.3% minerals by mass, respectively. The lean reference body had a physical tissue density of 1093 ± 7.1 kg m^−3^ and consisted of 69.1% ± 2.1% water, 27.4% ± 2.1% protein and 3.4% ± 0.4% minerals by mass. Predicted values of tissue density and lipid store corresponded well with independent empirical observations (Figure 3D,E).

### 3.3 Estimates of Lipid Stores

**FIGURE 4:**
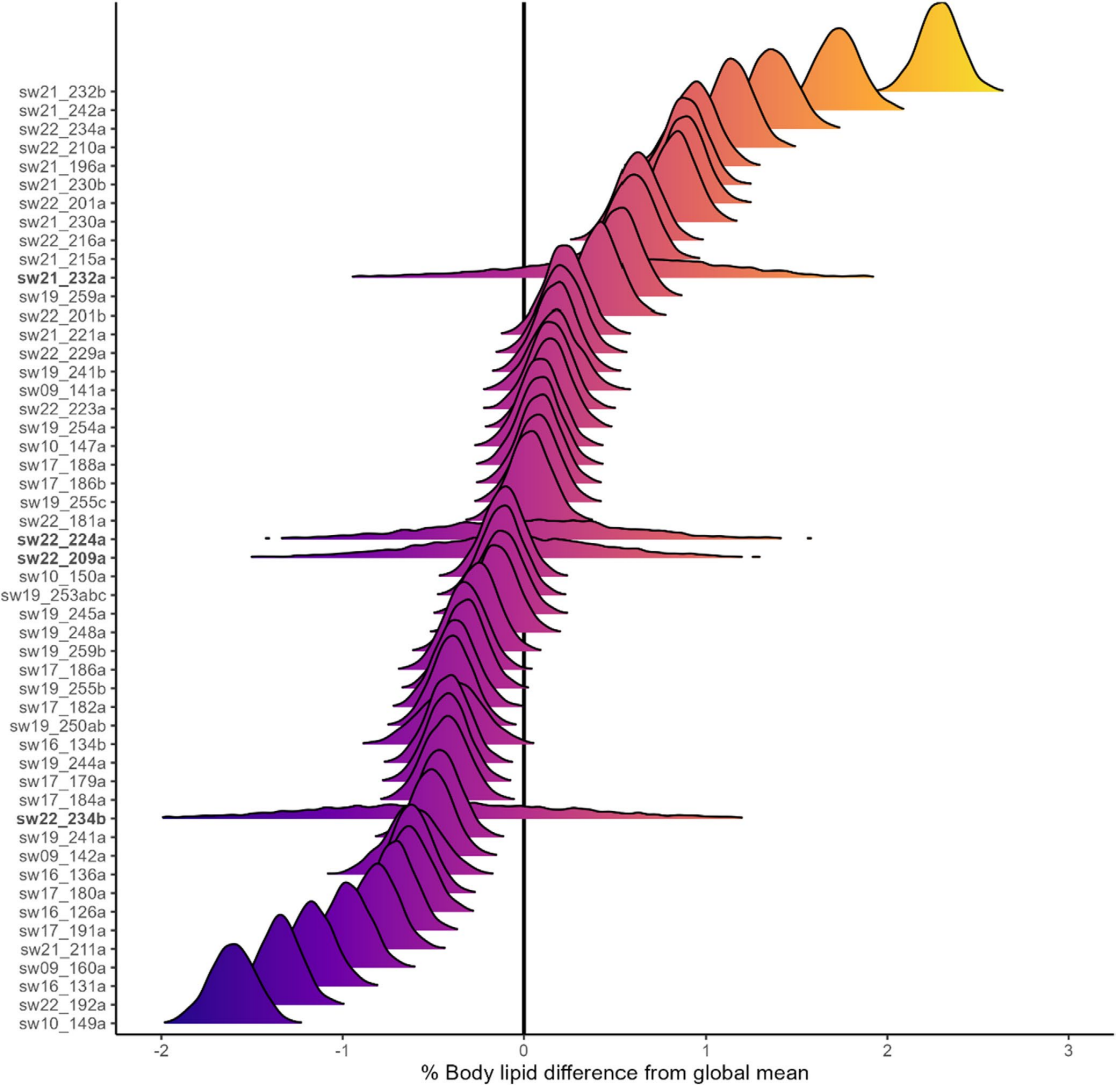
Estimated difference in tissue lipid % of each whale relative to a whale of global mean density. Identifiers in bold are whales for which only UAV observations were available and lipid proportions were therefore imputed by the Bayesian estimation framework.

**FIGURE 5:**
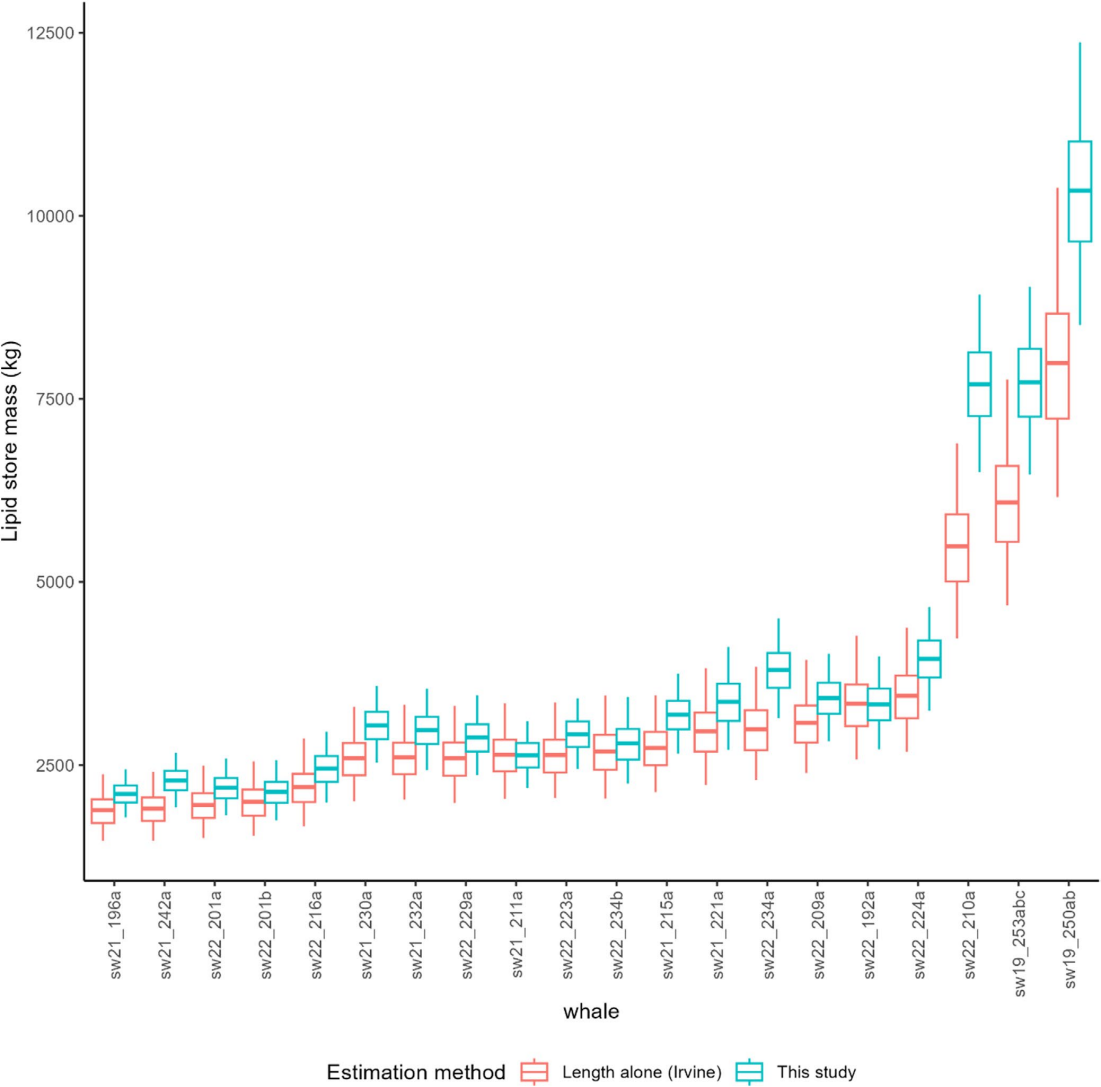
Estimates of absolute lipid store. Estimates are derived from model estimates of true underlying volume and density. Boxplots show median values ±50 and 95% quantiles (box and whiskers, respectively). Whales are sorted by increasing total length.

Posterior mean estimates of differences in lipid proportion relative to the global average ranged from 1.61% to 2.29% (Figure 4). As expected, posterior estimates from whales that lacked tissue density data directly informing lipid proportion (i.e., whales with UAV but not tag data; bold IDs in Figure 4, hollow points in Figure 2A) were less tightly constrained than those where tag‐derived tissue density was available. The posterior mean whale density corresponded to a body fat percentage of 22.5% ± 3.6%, estimated using Equation (5) and the lean reference distribution from the Monte Carlo simulations. Post hoc individual absolute lipid mass estimates ranged from 2.06 to 10.1 t and 1.7 to 7.8 t, with and without the spermaceti organ (Figure 5 and Table 3).

## 4 Discussion

Our primary aims in this study were to use simultaneous observations of sperm whale volume and tissue density (proxying composition) to test the hypothesis that they share a detectable underlying lipid store body condition driver, and to estimate the lipid store in free‐ranging sperm whales. These aims were accomplished using a combination of UAV image‐based volumetrics, densitometry based on tag‐derived tissue density and historical whaling data, and Bayesian hypothesis‐testing models. Statistical analysis supported a negative relationship between density and volume‐based indices of body condition, confirming that the two methods measure a common underlying body condition state. By using historical whaling data to convert density to tissue composition and combining the results with measurements of body volume, we were successful at estimating total body lipid mass to within an estimated 18% on average (calculated as the 95% CI divided by the estimate; Figure 5).

### 4.1 Sperm Whale Allometry

The allometric exponent for total body volume increased slightly from the prior mean (2.88 vs. 2.86, respectively) specified from Glarou et al. (2023), but remained less than three, indicating that sperm whale bodies become thinner relative to their length as they get larger. This suggests that scale‐invariant metrics such as length‐standardised surface area (Aoki et al. 2021; Christiansen et al. 2016) or body area index (Burnett et al. 2018) may be somewhat biased in this species. These metrics should therefore be avoided when comparing individual sperm whales which differ substantially in size. In other species, such metrics should only be adopted after it has been empirically demonstrated, using scaled imagery, that the allometric volume or area exponent is approximately 3 or 2, respectively (Christiansen et al. 2018; Glarou et al. 2023). Relative measurement imprecision varied considerably across whales and was larger on average when measuring whales than when measuring an object of known length (total length CV across‐whales range = 0.0014–0.039, mean = 0.018, Table 3 vs. 0.0033 across altitudes of 6.62–29.9 m, respectively) in support of our chosen statistical approach of estimating measurement uncertainty using repeated measurements of individual whales.

### 4.2 Body Condition Cross‐Validation

In this analysis we successfully validated two different methods of measuring body condition, morphometry and densitometry, in sperm whales. This result indicates firstly that lipid store‐body condition state drives both volume and density, and secondly that the respective methods used to quantify these metrics are sufficiently precise to detect changes in this state (coefficients of variation [CV, across dataset mean ± SD] UAV volume: 0.06 ± 0.03 [repeated measures], tissue density: 6.2 × 10^−5^ ± 2.7 × 10^−5^ [posterior samples]; also see Table 3). Specifically, we found a negative relationship, indicating that animals with a larger volume: length ratio also contained more fat per unit volume (and therefore lower tissue density, Figure 2). On average, for a whale 12 m in length, an increment in volume of 1 m^3^ was predicted to be accompanied by a 6.3% increase in total body lipid mass (including the predicted accompanying change in lipid percentage). The same 1 m^3^ increase in pure blubber would have predicted a 12.5‐14.4% increase (Lockyer 1991), emphasising that changes in morphometric condition may reflect changes in both lean and adipose tissue volume and the benefits of incorporating compositional information when calculating total body lipid store. The corresponding change in lipid mass for a decrease in tissue density of 1 kg m^−3^ was 5.1%.

### 4.3 Cost of Growth

Average estimated tissue composition corresponded to an energy density of 14.9 ± 1.32 kJ g^−1^ and a cost of growth of 23.32 ± 1.56 kJ g^−1^ assuming energy densities of 23.6 and 42.5 kJ g^−1^ (Koopman 2018; Lockyer 1991) and deposition efficiencies of 0.47 and 0.79 (Adamczak et al. 2023) for proteins and lipids respectively. Applying the mean posterior allometric function then predicts a cost of structural growth of roughly 24.0 GJ m^−3^ or 125.1 GJ m^−1^ for an animal that starts with a length of 11 m and a volume of 23.5 m^3^. These values align broadly with estimated embodied energy and costs of growth for other cetaceans (Adamczak et al. 2023). For the purposes of bioenergetic modelling, these estimates could be combined with ontogenetic growth curves to estimate annual costs of somatic growth.

### 4.4 Method Limitations, Transferability and Future Directions

Compared to equivalent predictions of sperm whale total body lipid content based on a length yield model fitted to the independent whaling dataset (Figure 5) the method developed here was broadly consistent and more precise (CVs 0.13 and 0.09, respectively), although it produced slightly higher estimates, particularly for the three large males. This is not unexpected, since while the yield values we used reflected processing of the whole body, this typically does not extract 100% of body lipids. Additional contributing factors may include small sample size (of both our sample and that of Glarou et al.'s height to width ratios [*n* = 7]) and lack of available side‐on imagery for large males. This necessitated generalising height: width ratios from smaller animals of unknown sex to large males (although note that model inference was unchanged by the exclusion of large males; Table S1.3.8). Additionally, such differences may arise from differences in lipid store allometry between the Pacific/Southern Ocean (location of whaling data) and Atlantic populations (where our measurements were taken).

When estimating absolute individual lipid proportion, uncertainty is a product of both tissue density observation error and reference body uncertainty. As with humans (Siri 1956), uncertainty attributable to the reference body was comparable in magnitude to likely observation error, indicating that material improvements in prediction could be achieved by reducing uncertainty associated with the reference body, which can arise from inherent underlying biological variability or more tractable uncertainties arising from measurement error and data gaps. Analysis of whaling data indicated that, as expected, uncertainty surrounding lean body density was low compared with that for the whole body (Figure 3D). Average lipid store, in the blubber and elsewhere, is likely to vary systematically between individuals and populations depending on stored energy, limiting transferability. On the other hand, the composition and resultant density of lean tissues such as muscle, bone, blood and vital organs appeared much less variable (Figure 3B,C), probably because they are more tightly constrained by function. True variability in lean tissue composition is probably relatively low among marine mammal individuals and species, which share an aquatic environment and many resulting functional demands and trade‐offs (Adamczak et al. 2023). It is likely that the much of the imprecision in the present sperm whale lean density estimate results from measurement error and conservatism in the model formulation, rather than genuine biological variability in lean tissue composition, and that there is scope for considerable improvement before the precision ceiling imposed by the latter is approached.

Ultimately, the only way to confirm the accuracy (rather than precision) of the methods presented in this study would be through direct validation on individual whales. This could potentially be accomplished by studying strandings, for example, by imaging animals which appear at risk of stranding, then measuring the tissue volume and composition and/or tissue density of individuals which then go on to strand (for a case study see Currie et al. 2021).

The fitted model and code are freely available and can be used to impute plausible values of total body lipid volume for additional UAV only observations of sperm whales (and other species if suitable volume and density datasets and reference body densities are supplied), allowing robust lipid store estimation with minimal disturbance compared to tagging. Conversely, the model could be used to improve on‐animal estimates of lipid store from density estimating biologging tags (Adachi et al. 2023) by incorporating the increases in body volume given length that are predicted to accompany observed decreases in density.

The predictive power of the modelling approach is maximised by supplying as many concurrent observations of density, length and volume as is practical. While simultaneous tag and drone observations have historically been challenging to collect, more recently UAVs have begun deploying biologging tags on free‐ranging cetaceans to minimise disturbance to the target animal during tagging (Wiley et al. 2023). Using this approach, the collection of contemporaneous overhead photogrammetry could be embedded in standard tagging practice, maximising the value of the resulting data for health assessment with minimal additional effort simply by ensuring that tagging UAVs are equipped with suitable altimeters and low‐distortion optics.

While our method is well suited to large cetaceans, it is potentially transferable to any swimming taxa for which tissue density can be measured (directly or by bearing suitable tags) and is available to scaled photogrammetry. Large sharks, for example, are commonly tagged using suitable tri‐axial movement logging (e.g., CATS) tags and are available to scaled photogrammetry either at the surface using UAVs or underwater using parallel‐laser photogrammetry. We envision therefore that the methods presented here may be readily applicable to these and other large aquatic taxa.

## 5 Conclusions

Our results confirm that there is a shared lipid‐store body condition driver of body volume and density in sperm whales and that the methods used are sufficiently precise to detect it. The shared component allows improved prediction of lipid store where only volume or density data are available, and the combined approach developed here allows for estimation of absolute lipid store to within an estimated 18% on average. While here the method is applied to sperm whales, it is potentially transferable to any swimming taxa which are available to scaled photogrammetry and tagging.

## Author Contributions

**Alec Burslem:** conceptualization (equal), data curation (equal), formal analysis (lead), investigation (lead), methodology (lead), project administration (equal), resources (equal), software (equal), validation (equal), visualization (equal), writing – original draft (lead), writing – review and editing (equal). **Saana Isojunno:** conceptualization (equal), data curation (equal), formal analysis (equal), investigation (equal), methodology (equal), project administration (equal), resources (equal), supervision (equal), validation (equal), visualization (equal), writing – review and editing (equal). **Rui Prieto:** data curation (equal), investigation (equal), methodology (equal), project administration (equal), resources (equal), writing – review and editing (equal). **Mónica A. Silva:** data curation (equal), funding acquisition (equal), investigation (equal), methodology (equal), project administration (equal), resources (equal), writing – review and editing (equal). **Patrick J. O. Miller:** conceptualization (equal), data curation (equal), formal analysis (equal), funding acquisition (equal), investigation (equal), methodology (equal), project administration (equal), resources (equal), supervision (equal), validation (equal), visualization (equal), writing – review and editing (equal).

## Conflicts of Interest

The authors declare no conflicts of interest.

## Supporting information


**Appendix S1:** ece372472‐sup‐0001‐AppendixS1.docx.

## Data Availability

Data, analysis code and additional model diagnostics underlying this paper are available in the Digital Data Supplement — DS1 hosted by the open science framework (OSF; Repository URL: https://doi.org/10.17605/OSF.IO/BUH2X).
