# Supplementary material for: Estimating Total Body Lipid Store of Free‐Ranging Whales In Vivo Using Drone Photogrammetry and Biologging Tags
Source: Ecol Evol. 2025 Nov 26;15(12):e72472. doi: 10.1002/ece3.72472 (PMC12657101; doi:10.1002/ece3.72472)
Supplement: Supplementary file 1 — Appendix S1: ece372472‐sup‐0001‐AppendixS1.docx. [file ECE3-15-e72472-s001.pdf]

Appendix S1 to '*Estimating total body lipid store in-vivo for free-ranging whales using drone photogrammetry and biologging tags*'

Alec Burslem[1]✉, Saana Isojunno[2], Rui Prieto[3], Mónica A. Silva[3] & Patrick J Miller[1]

1 Sea mammal research unit, Scottish Oceans Institute, University of St Andrews, St Andrews, Fife, UK, KY16 8LB
2 Centre for research into environmental and ecological modelling, University of St Andrews, The Observatory, Buchanan Gardens, University of St Andrews
3 Okeanos, University of the Azores, & Institute of Marine Research – IMAR, Horta, Portugal
✉ Alec Burslem, aburslem@hawaii.edu; Hawaii Institute for Marine Biology, 46-007 Lilipuna Rd, Kaneohe, Hawai'i, USA

Contents

S1.1. Additional details on data collection & processing 2
S1.2. Physics underlying the method: full derivations & additional details 4
S1.3. Development, specification and sensitivity of the statistical model: additional technical details 7
S1.4. Supplementary tables 22
S1.5. Supplemental figures 29
References 33

S1.1. Additional details on data collection & processing

Tagging

Sperm whales were tagged opportunistically as encountered, the only animals which were not targeted were calves and, in the Azores, whales we knew we had recently tagged, usually because it was bearing a tag, but occasionally from photo ID. Tags were applied by two methods, depending on the research vessel used. For larger vessels (e.g. '*Pintado*' a 10m rigid hulled inflatable boat [RHIB] used in the Azores), tags were applied from directly behind the whale using a 12m cantilevered carbon fibre pole to avoid encroachment of the vessel over the animal's tail fluke. From smaller vessels, which lacked a mount for the cantilever system, tags were applied using a 7m counterweighted carbon fibre hand-pole.

UAV photogrammetry

A DJI phantom 4 Pro UAV was used for all flights performed in the Azores in 2021-2, while a DJI phantom 4 was used for flights in Norway in 2019. Overhead photogrammetry images of sperm whales were obtained by flying the UAV above the animals at an elevation of 13-23 meters in winds not exceeding 6 m/s and sea state not exceeding Beaufort 3. The camera was operated using DJI Pilot software (DJI, 2020).

Both UAV platforms record altitude barometrically which is not always ideal for photogrammetry as it doesn't directly measure range to the ocean surface, so does not account for changes in sea surface height due to swell or tidal movements. A custom-built laser altimeter and datalogger package (Kelp Marine) was therefore fitted as additional payload to the UAVs to measure range from the UAV to the sea surface. In 2019, this system was configured to sample time and range at a rate of 1Hz. A real time clock (RTC) board on the datalogger allowed accurate time to be kept with a frequency stability of ± 2 ppm, which corresponds to a drift rate of around 1 second every 6 months. The RTC clock was set at the outset of the fieldwork and updated every two weeks thereafter. Pitch and roll of the UAV were recorded by the Phantom 4's onboard IMU and microprocessor, telemetered to the tablet and offloaded at the end of each day's flying. Video was captured in 4K resolution, allowing the entire surfacing sequence to be captured and suitable photogrammetry frames isolated later. Before each flight, an image taken of a handheld GPS device screen using the UAV camera was used to calculate the offset between accurate GPS time and the UAV system time, which was imprinted in the image and video EXIF data. The GPS device was also kept in view for at least one second roll-over after UAV video capture was started to independently synchronise the video frame timeseries with the GPS device. Video was then recorded continuously for the duration of the flight. Images were extracted and and matched to the laser altimeter and IMU timeseries using a custom written MATLAB script. Unless otherwise stated (see below) only frames with a valid laser altimeter reading taken within one second and meeting established quality criteria (Christiansen et al., 2018) were used.

For the 2021 and 2022 Azores field seasons, the system was upgraded. For these years we switched UAV platforms to the DJI phantom 4 Pro (P4P), which has lower lens distortion and

longer flight endurance than the P4. The laser altimeter dataloggers were also upgraded to sample at a higher high frequency (5 vs 1 Hz; Kelp Marine) and fitted with tilt sensors and a GPS board. This system was able to record pitch, roll and LiDAR data streams at 5hz with GPS quality timestamps, requiring only a single synchronisation step with the video time series.

In the one case where the laser altimeter unit failed completely (likely due to a loose connection, whale SW21_230b), the UAV's onboard barometric altitude was used. Inaccuracies with barometric altitudes tend to be largely a result of bias with respect to the origin (which is initialised at take-off) rather than measurement imprecision, and have been shown to give acceptable accuracy for marine mammal photogrammetry when a suitable offset between sea level and altitude at take-off is given (Bierlich et al., 2021; Burnett et al., 2018). *Pintado* floats with her deck level with the waterline; we therefore estimated range to sea surface in this case by measuring the height of coauthor RP with his arms upstretched in the position he launched the drone and added this known value to the barometric altitude time series.

Both photogrammetry systems used the Lightware Laser SF11C altimeter, with a manufacturer rated accuracy of ± 10cm (LightWare Optoelectronics, 2018). We also conducted additional checks of the accuracy of both photogrammetry systems. For the P4 system (fixed focal length), the altimeter's accuracy was double checked by measuring a known distance (mean error = 2.46cm or 0.5%, SD = 2.19cm at 4.57m). To account for the variable focal length of the Phantom 4 pro we flew a calibration flight measuring the same object of known length over a range of heights and fitted an empirical calibration model to the resulting data. As expected, we found a systematic relationship between height and measurement error ($p < 10^{-6}$), resulting from changes in focal length required to keep the object in focus (although not expected, any altimeter bias would also have been represented in the same model term). After focal lengths were corrected using the fitted model, the length of the object was estimated from to within 0.9 ±0.28cm (mean absolute error ± 95% CI) or 0.3 ± 0.1% across a range of altitudes encompassing those of our field measurements (6.2-25.9m). Our field measurements were therefore adjusted using this model. As a final check of accuracy *in-situ*, in a subset of images where the tag placement was approximately flat and level on the back of the whale, we also took measurements of the tag on the whale. This showed no evidence that appreciable systematic bias (i.e. mean measurement error significantly different from 0) remained for either the phantom 4 (one sample t test p = 0.27, mean absolute error = 1.2 ± 3.0cm) or phantom4 pro system (p = 0.52, mean absolute error = 1.4 ± 3.2cm), including for a separate analysis of the flight where barometric altitude had to be used (p = 0.5, mean absolute error = 1.0 ± 1.30cm).

S1.2. Physics underlying the method: full derivations & additional details

Density, volume and proportions by mass of a compartmentalised body

The relationship between volume (V) and density (ρ) of an n-compartment body, each compartment with its own separate density, can be stated as:

$$\rho = \frac{\sum_{c=1}^{n} \rho_c \cdot V_c}{V}$$

(eqn 1, main text)

To express the relationship in terms of proportions by mass of each compartment (P_c) which sum to one, i.e.,

$$P_c = \frac{\rho_c \cdot V_c}{\rho \cdot V}$$

(eqn S1)

and given the volume of each compartment must also sum to one, we can substitute:

$$1 = \sum_{c=1}^{n} \frac{V_c}{V},$$

(eqn S2)

divide through by ρ:

$$\frac{1}{\rho} = \sum_{c=1}^{n} \frac{V_c}{\rho \cdot V},$$

multiply by $1 = \frac{\rho_c}{\rho_c}$:

(eqn S.3)

$$\frac{1}{\rho} = \sum_{c=1}^{n} \frac{V_c \cdot \rho_c}{\rho \cdot V \cdot \rho_c},$$

(eqn S.4)

and, simplifying with Eq. S1, we arrive at the following expression:

$$\frac{1}{\rho} = \sum_{c=1}^{n} \frac{P_c}{\rho_c}.$$

(eqn S5)

Thus, if the overall density of the body, the density and proportional masses of n-1 compartments (either separately or combined) and the density of the final compartment (ρ_n) are known, it is possible to solve for the proportional mass of the final compartment analytically:

$$P_n = \rho_n \cdot \left(\frac{1}{\rho} - \sum_{c=1}^{n-1} \frac{P_c}{\rho_c}\right).$$

(eqn 2, main text)

Densitometry

To solve for the proportion of an animal's body mass that consists of lipid (hereafter, lipid proportion, P_l), given an observed tissue density (ρ_t) and a known lipid density (ρ_l), it is necessary to specify a reference body (γ). The reference body is specified a lipid proportion ($P_{l\gamma}$) to which some proportional energy storage component (τ), is assumed to have been added (or removed). The reference body can take the form of an idealised average body, such as. the 'standard man' used in early human densitometric studies (Keys and BroŽek, 1953; Siri, 1956) which is specified some constant proportion of lipid $P_{l\gamma}$ and density ρ_γ. Alternatively, the reference body may be specified to only comprise lean tissue, in which case $P_{l\gamma} = 0$ and only lean density (ρ_ζ) needs to be known.

The equation to calculate the total body lipid proportion from a given tissue density (ρ_t) and reference body γ takes the following general form (Siri, 1956; Eq.7 therein):

$$P_l = \frac{\rho_\tau \cdot \rho_\gamma}{\rho_t} \cdot \left(\frac{P_{l\tau} - P_{l\gamma}}{\rho_\gamma - \rho_\tau}\right) - \frac{\rho_\tau \cdot P_{l\tau} - \rho_\gamma \cdot P_{l\gamma}}{\rho_\gamma - \rho_l}$$

(eqn 3)

Specifying the expected values for tissue density and lipid proportion ($\overline{\rho_t}$ and $\overline{P_l}$) as those of the reference body (ρ_γ and $P_{l\gamma}$, respectively), the idealised average whale body (the 'standard whale') can be defined as the reference body. Then, specifying the density of the energy store (ρ_τ) to be the density of lipid (ρ_l), and setting the proportion of reserves consisting of lipid to one ($P_{l\tau} = 1$), we can express Eq. 3 as:

$$P_l = \frac{\rho_l \cdot \overline{\rho_t}}{\rho_t} \cdot \left(\frac{1 - \overline{P_l}}{\overline{\rho_t} - \rho_l}\right) - \frac{\rho_l - \overline{\rho_t} \cdot \overline{P_l}}{\overline{\rho_t} - \rho_l}$$

(eqn S6)

where pure lipid and the average body are taken as the variable reserve component τ and reference body γ, respectively.

Alternatively, without specifying the lipid proportion of the reference body $P_{l\gamma}$, we can still solve for the difference in lipid proportion (δP_l) between the body and the reference body ($\delta P_l = P_{l[w]} - P_{l\gamma}$):

$$\delta P_l = \frac{1}{\rho_t} \cdot \left(\frac{\rho_\gamma \cdot \rho_\tau}{\rho_\gamma - \rho_\tau} \right) - \frac{\rho_\tau}{\rho_\gamma - \rho_\tau}$$

(eqn 4)

(Siri, 1956, Eq.6 therein). Therefore, specifying the reference body to only be comprised of lean tissues with a known density ρ_ζ, we can express the lipid proportion by setting $\rho_\gamma = \rho_\zeta$ and, again equating energy and lipid stores ($P_{l\tau} = 1$, $\rho_\tau = \rho_l$):

$$P_l = \delta P_l = \frac{1}{\rho_t} \cdot \left(\frac{\rho_\zeta \cdot \rho_l}{\rho_\zeta - \rho_l} \right) - \frac{\rho_l}{\rho_\zeta - \rho_l}.$$

(eqn 5)

In comparison with the standard whale reference body (Eq. S6), the lean reference body (Eq. 4.15) offers a simpler equation and assumes only one unmeasured constant: lean density (ρ_ζ), which is expected to be less variable among whales and populations than expected lipid proportion (P_l). This form (Eq. 4.15) was therefore preferred *a priori.*

A third alternative approach would be to specify a structural mass reference body. This approach would explicitly account for the fact that there is some level of lipid store in the body that is essential for function and so should not be thought of as part of the lipid energy store. This approach could potentially be preferable in a better-understood system, but necessitates specifying a minimum value of structural lipid necessary for the functioning of each tissue compartment (Ellis, 2000). We opted not to pursue this approach, given the uncertainty about the functional role of lipids, and particularly wax esters, in the bodies of sperm whales, and the inability of densitometric methods to distinguish between lipid classes. Instead, likely lipid store subdivisions and their potential energetic and functional implications were explored post-hoc.

S1.3. Development, specification and sensitivity of the statistical model: additional technical details

Model specification

The model was specified a hidden process for underlying body length and tissue density (Eqs. S7-S11) and an observation process for the measured tissue density, body length, and body volume (Eqs. S12-S17). The model was specified to estimate underlying body volume as a function of whale length and density (Eqs. S9-S11) and identify individual differences in lipid percentage from the average by treating the global expectation of tissue density as the reference body within the model. This model thus simultaneously tested the hypothesis that tissue density and residual volume share body condition as an underlying driver and estimated how much each whale differs from the average in units of body fat percentage. This approach allowed the model to make fewer assumptions compared to a model specifying lean density estimates as the reference body density, which would have led to identifiability problems unless fixed at a single point value. Lipid density still had to be set to a fixed value but, unlike lean density, has been measured empirically for sperm whales at body temperature, varies only negligibly between tissues (R. Clarke, 1978; Lonati *et al.*, 2019, Table S1.3.1) and is therefore not expected to affect model inference.

Table S1.3.1: Measured tissue constituent densities

The value for lipid density ρ_l represents the density of an 11:89 mixture of pure spermaceti and blubber lipid at body temperature.

Parameter	Value (kg m^{-3})	Source (s)
$\boldsymbol{\rho_l}$	857.228	(Clarke, 1978b; Lonati et al., 2019, Table 3 therein)
$\boldsymbol{\rho_p}$	1340	(Moore et al., 1963)
$\boldsymbol{\rho_w}$	994	(Moore et al., 1963)
$\boldsymbol{\rho_a}$	2300	(Moore et al., 1963)

Measurement error was specified at the level of individual whales, allowing less certain observations of length, volume and tissue density in the model to exert less influence on the linear predictor than more certain ones. Tissue density measurement error was specified as data and corresponded to the posterior tissue density error output by the upstream hydrodynamic modelling framework. Stochastic measurement error associated with UAV measurements of whale length and volume observations, on the other hand, was estimated within the model using repeated observations of the same whale taken within the same day

The model did not include diving gas volume (V_g) explicitly, but this is not expected to influence body condition metrics. The hydrodynamic modelling method uses glide acceleration at various depths to estimate diving gas volume and tissue density terms separately. Body volume was measured at the surface, so it included the volume of both tissue and the air in the lungs. However, changes in air volume due to tidal breathing do not cause detectable within-surfacing

changes in UAV measurements of body width (Glarou et al., 2023). Air volume should therefore only lead to the model selecting a slightly different value for the log intercept (α_V) and therefore expected volume in the model, while residual volumes should be unaffected.

Informative priors were specified as shown in the main text (Table 2) and the full model specification is given as NIMBLE code below.

Hidden process for individual whales w

$$L_{u[w]} \sim Gamma\left(shape = \left(\frac{\overline{L_u}}{\sigma_{Lu}}\right)^2, rate = \frac{\overline{L_u}}{\sigma_{Lu}^2}\right)$$

(eqn S7)

$$\rho_{u[w]} \sim Gamma\left(shape = \left(\frac{\overline{\rho_u}}{\sigma_{\rho u}}\right)^2, rate = \frac{\overline{\rho_u}}{\sigma_{\rho u}^2}\right)$$

(eqn S8)

$$\delta P_{l[w]} = \frac{1}{\rho_{u[w]}} \cdot \left(\frac{\overline{\rho_u} \cdot \rho_l}{\overline{\rho_u} - \rho_l}\right) - \frac{\rho_l}{\overline{\rho_u} - \rho_l}$$

(eqn S9)

$$log\left(\overline{V_{u[w]}}\right) = log(\alpha_V) + log\left(L_{u[w]}\right) \cdot \beta_V$$

(eqn S10)

$$log\left(V_{u[w]}\right) = log\left(\overline{V_{u[w]}}\right) + \beta_{\delta P_l} \cdot \delta P_{l[w]}$$

(eqn S11)

Observation process for individual whales w and images i

$$\sigma_{Lo[w]} \sim Gamma\left(shape = \left(\frac{\overline{\sigma_{Lo}}}{\sigma_{\sigma Lo}}\right)^2, rate = \frac{\overline{\sigma_{Lo}}}{\sigma_{\sigma Lo}^2}\right)$$

(eqn S12)

$$\sigma_{Vo[w]} \sim Gamma\left(shape = \left(\frac{\overline{\sigma_{Vo}}}{\sigma_{\sigma Vo}}\right)^2, rate = \frac{\overline{\sigma_{Vo}}}{\sigma_{\sigma Vo}^2}\right)$$

(eqn S13)

$$\sigma_{\rho o[w]} \sim Gamma\left(shape = \left(\frac{\overline{\sigma_{\rho o}}}{\sigma_{\sigma \rho o}}\right)^2, rate = \frac{\overline{\sigma_{\rho o}}}{\sigma_{\sigma \rho o}^2}\right)$$

(eqn S14)

$$\rho_{o[w]} \sim Gamma\left(shape = \left(\frac{\rho_{u[w]}}{\sigma_{\rho o[w]}}\right)^2, rate = \frac{\rho_{u[w]}}{\sigma_{\rho o[w]}^2}\right)$$

(eqn S15)

$$L_{o[i,w]} \sim Gamma\left(shape = \left(\frac{L_{u[w]}}{\sigma_{Lo[w]}}\right)^2, rate = \frac{L_{u[w]}}{\sigma^2_{Lo[w]}}\right)$$

(eqn S16)

$$log\left(V_{o[i,w]}\right) \sim dnorm\left(mean = log\left(V_{u[w]}\right), sd = \sigma_{Vo[w]}\right)$$

(eqn S17)

Equation S8 treats the global average as the reference body, the lipid proportion of which is unknown. Equation S8 was obtained from Eq. 4 by substituting the reference body ρ_τ with the underlying global average $\overline{\rho_u}$, equating lipid with energy store ($P_{l\tau} = 1$, $\rho_\tau = \rho_l$) and setting $\rho_{t[w]} = \rho_{u[w]}$. This expression therefore solves for the difference in lipid proportion $\delta P_{l[w]}$ between whale *w* and a whale with the estimated global average density at each update of the MCMC. As it is a proportion, it is incorporated as an additional linear predictor of the log (and therefore multiplicative) volume residuals (Eq. S11) with no log transformation.

Model testing and sensitivity checks

To test whether the model was correctly specified and capable of recovering unbiased parameter values, we ran several test models on simulated data (Table S1.3.2). Data were simulated using equations corresponding to the process model assumed by the specified likelihood (Eqs. S7-S17) and plausible 'true' values for the estimable parameters. Observation and process errors were specified based on previous empirical analyses and characteristics of the observed dataset (Evans et al., 2003; Glarou et al., 2023; Miller et al., 2016).

The full model as specified reproduced approximations of all model parameters when fitted to a representative simulated sample (simulated sample sizes: volume: 10, tissue density: 30, both: 20): the 95% credible intervals encompassed the true value for each estimable parameter ('Correct' run, Table S1.3.3). Estimates of whale density ρ_u with larger observation error were pulled more strongly away from their true values, demonstrating that they exerted less influence on the fitted model (Fig. S1.3.1). This pattern was less obvious for V_u, possibly because volume observation error σ_{vo} was estimated rather than specified as data so was itself more uncertain.

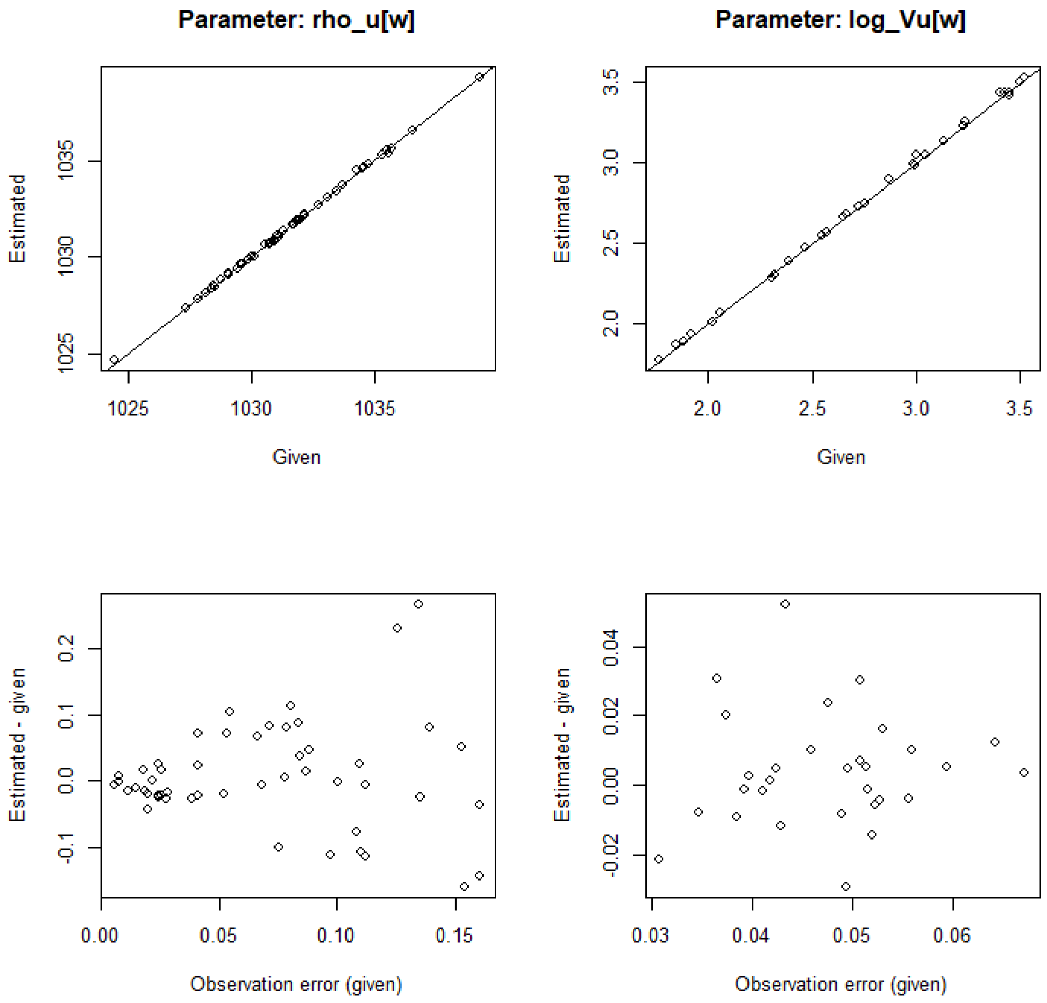

Figure S1.3.1. Unbiased parameter recovery diagnostic plots for the full model fitted to a representative simulated dataset ('correct' run, Table S1.3.2)

Upper panels show given values used to simulate the dataset (x axis) vs model estimates. Diagonal line is the line of equality (i.e. perfect estimation). Bottom panels show the distance the estimate was 'pulled' from its true given value (y axis) as a function of observation error (x axis). Note the funnel shape in the bottom left-hand plot, indicating that more uncertain values are pulled more strongly towards the expectation.

The allometric parameters α_v and β_v showed poor mixing due to lack of identifiability (Figs. S.1.3.2-1.3.3) when specified uninformative priors (Table S1.3.4). This was likely due to the small range in length in the observed data and the relatively gentle curve in the expected allometric relationship, i.e. almost identical length to volume curves can be realised by multiple combinations of linear and exponential coefficients. This issue was resolved by specifying informative priors based on Glarou *et al.*'s (2023) results. As expected, these strong priors made the model susceptible to mismatch between the priors for allometry parameters β_{vu} and α_{vu} and the values inferred by the data. When the given 'true' values of the simulated data were very

different from the priors specified, the β_{vu} and α_{vu} parameters were biased towards the priors (Table S1.3.5). Such a strong mismatch is not expected, but the priors (and/or H:W ratios) could conceivably be unrepresentative for large males, which were not present in Glarou *et al.*'s sample, and could therefore potentially bias the estimates of residual volume, affecting inference.

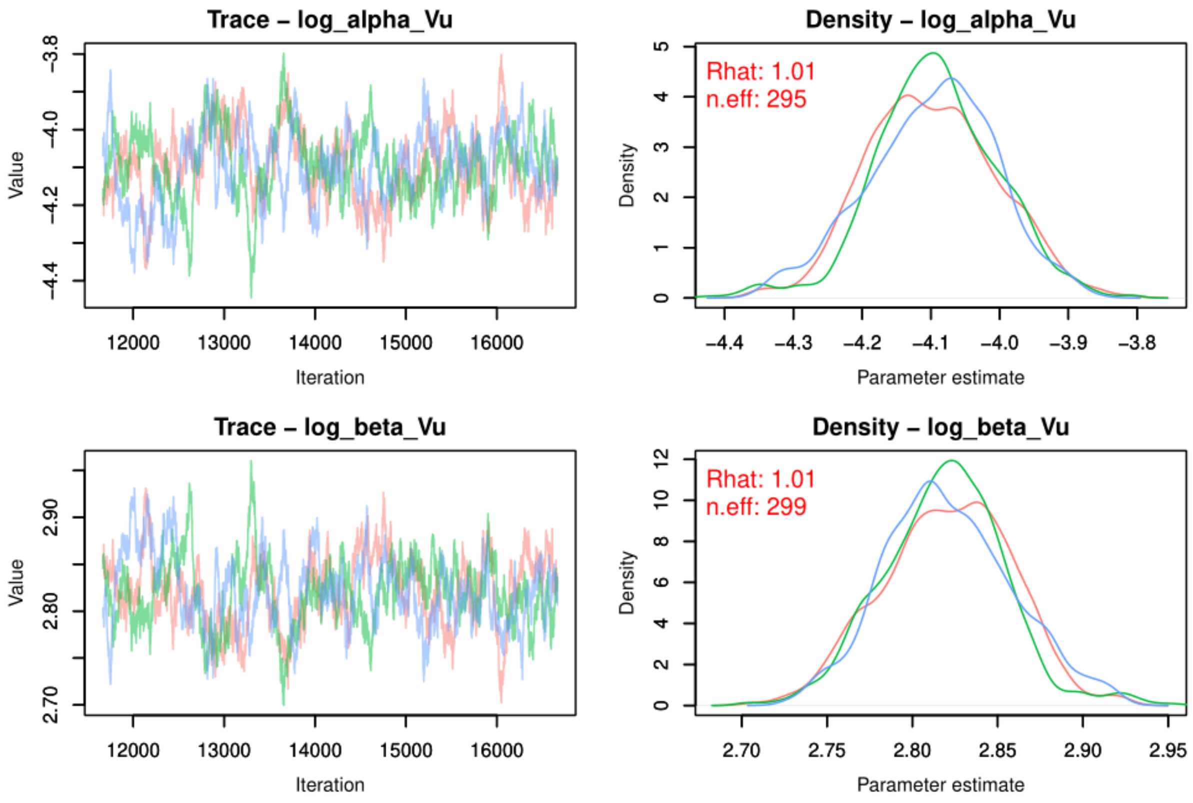

Figure S1.3.2. Example posterior MCMC trace and density plots

These traces are from the 'uninformative' test model (Tables S1.3.2 and S1.3.4) MCMC and were used to diagnose slow mixing of the Markov chains for these parameters.

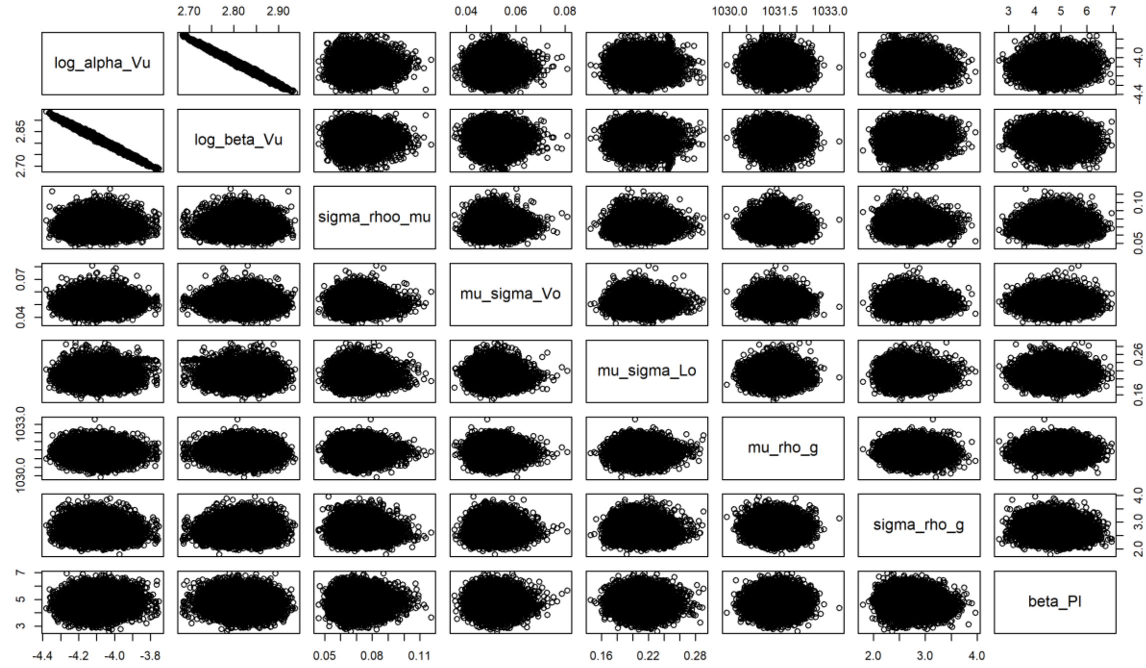

Figure S1.3.3 Example posterior samples diagnostic plot assessing identifiability for global parameters

This plot is from the 'uninformative' test model (Tables S1.3.2 and S1.3.4) MCMC and was used to diagnose poor identifiability between log_beta_Vu (β_V) and log_alpha_Vu (α_V) as the likely cause of the slow mixing of the Markov chains for these parameters (Figure S1.3.2).

We therefore opted to fit the full model to the real data and to fit an additional model to check that the hypothesis test result was not a result of the strong priors overwhelming the inference from the data. For this additional model (Check1), the residual volume observations were calculated outside of the statistical model. This was accomplished by fitting an ordinary least squares model of log volume and length to the best quality image for each whale. The predictions of this model for the full dataset of observed lengths were then subtracted from the observed log volumes. This approach estimated the log-volume residuals directly for the present dataset and removed the between-image variability in estimated volume which is purely a function of differences in estimated length, while leaving that which is a product of body condition or volume observation error. The resulting body condition metric (BC, Christiansen *et al.*, 2018; Glarou *et al.*, 2023) can be conceptualised as the three-dimensional equivalent of a two-dimensional length standardized metric (e.g. LSSAI, Aoki *et al.*, 2021) but with an empirical, rather than assumed, scaling exponent. A simplified model with no allometric parameters (see Check1 model jags code below) was fitted to these data. This approach was successful in recovering true parameters from simulated data without the need to specify allometric parameters (Table S1.3.6). As a final sensitivity check, we fitted the full model to the observed data with the large males removed from the dataset. Both the check1 model and the full model fitted to observed data with the large males removed also estimated a positive value for $\beta_{\delta l}$

(posterior means = 7.62 and 6.01, 95% CI ranges = 5.3-10.1 and 1.5-10.5, respectively; Tables S1.3.7 and S1.3.8), in agreement with the full model.

Full model

```
  #global

  # allometry
  log_alpha_Vu ~ dnorm(mean = -4.04793 , sd = 0.08646) #
Glarou et al
  log_beta_Vu ~ dnorm(mean = 2.86083, sd = 0.04094) # Glarou
et al
  Lu_mu ~ dunif(1e-06, 17)
  Lu_sigma ~ dunif(1e-06, 100)

  # density observation error
  sigma_rhoo_mu ~ dunif(1e-06,100)
  sigma_rhoo_sigma ~ dunif(1e-06,100)

  # global average volume observation error
  mu_sigma_Vo ~ dunif(1e-06,100)
  sigma_sigma_Vo ~ dunif(1e-06,100)
  mu_sigma_Lo ~ dunif(1e-06,100)
  sigma_sigma_Lo ~ dunif(1e-06,100)

  mu_rho_g ~ dgamma(mean = 1030, sd = 14.5) # Miller et al
2004
  sigma_rho_g ~ dunif(1e-06,100)

  beta_Pl ~ dnorm(0, sd = 50) # conservative prior

  # whale-level priors/likelihood
  for(w in 1:NW){
    #process
    L_u[w] ~ dgamma(mean = Lu_mu , sd = Lu_sigma)
    rho_u[w] ~ dgamma(mean = mu_rho_g, sd = sigma_rho_g)
    delta_Pl[w] <- (1/rho_u[w]) * ((mu_rho_g *
rho_l)/(mu_rho_g - rho_l)) - (rho_l / (mu_rho_g-rho_l))
    log_V_u.mu[w] <- log_alpha_Vu + log(L_u[w])*log_beta_Vu #
body volume expectation
    log_V_u[w]  <- log_V_u.mu[w] + beta_Pl * delta_Pl[w] #
true body volume

    #observations
    sigma_Lo[w] ~ dgamma(mean = mu_sigma_Lo, sd =
sigma_sigma_Lo)
    sigma_Vo[w] ~  dgamma(mean = mu_sigma_Vo, sd =
sigma_sigma_Vo)
    #density
    sigma_rhoo[w] ~ dgamma(mean = sigma_rhoo_mu, sd =
sigma_rhoo_sigma) # density observation error
    rho_o[w] ~ dgamma(mean = rho_u[w], sd = sigma_rhoo[w]) #
observed density
```

```
    }
  # observation level
  for(i in 1:N){
    # images
    log_V_o[i] ~ dnorm(mean = log_V_u[whale[i]], sd =
sigma_Vo[whale[i]])
    L_o[i] ~ dgamma(mean = L_u[whale[i]], sd =
sigma_Lo[whale[i]])
    #
  }
```

Check1 model

```
  # global priors

  # density observation error
  sigma_rhoo_mu ~ dunif(1e-06,100)
  sigma_rhoo_sigma ~ dunif(1e-06,100)


  alpha_BC ~ dnorm(0, 0.11) # glarou residuals
  mu_sigma_BC ~ dunif(1e-06,100)
  sigma_sigma_BC ~ dunif(1e-06,100)

  mu_rho_g ~ dgamma(mean = 1030, sd = 14.5) # Miller et al
2004
  sigma_rho_g ~ dunif(1e-06,100)

  beta_Pl ~ dnorm(0, sd = 50) # conservative prior

  # whale-level priors/likelihood
  for(w in 1:NW){
    #process
    rho_u[w] ~ dgamma(mean = mu_rho_g, sd = sigma_rho_g)
    delta_Pl[w] <- (1/rho_u[w]) * ((mu_rho_g *
rho_l)/(mu_rho_g - rho_l)) - (rho_l / (mu_rho_g-rho_l))
    BC_u[w] <- alpha_BC + beta_Pl * delta_Pl[w]  # true vol
resid.
    #observations
    sigma_BC[w] ~  dgamma(mean = mu_sigma_BC, sd =
sigma_sigma_BC)
    #density
    sigma_rhoo[w] ~ dgamma(mean = sigma_rhoo_mu, sd =
sigma_rhoo_sigma) # density observation error
    rho_o[w] ~ dgamma(mean = rho_u[w], sd = sigma_rhoo[w]) #
observed density
  }
  # observation level
  for(i in 1:N){
    # images
    BC_o[i] ~ dnorm(mean = BC_u[whale[i]], sd =
sigma_BC[whale[i]])
    #
  }
```

Table S1.3.2. Names and specification of test models fitted to simulated data

Model: model specification. Priors are set as stated in the main text (Table 2) unless stated otherwise. Given 'true' values used to simulate the data are shown in Tables S1.3.3-S1.3.6.

Name	Prior vs given value mismatch?	Model
**Correct**	Weak	Full
**Mis -specified**	Strong	Full
**Uninformative**	No informative priors	Full
**Check1**	No informative priors	Check1

Table S1.3.3. Estimated vs given parameter values for the 'Correct' run

Mean and 95%CI refer to the posterior estimates, while given refers to the 'true' parameter values used to simulate the dataset. Priors are set as stated in the main text (Table 2).

	Mean	95%CI_low	95%CI_upp	given
**log_alpha_Vu**	-4.09	-4.19	-3.99	-4.04
**log_beta_Vu**	2.82	2.78	2.86	2.80
**sigma_rhoo_mu**	0.06	0.05	0.07	0.06
**sigma_rhoo_sigma**	0.04	0.03	0.05	0.04
**sigma_sigma_Vo**	0.01	0.00	0.02	0.01
**mu_sigma_Vo**	0.05	0.05	0.06	0.05
**sigma_sigma_Lo**	0.05	0.00	0.09	0.01
**mu_sigma_Lo**	0.20	0.17	0.23	0.20
**mu_rho_g**	1030.27	1029.46	1031.08	1030.00
**sigma_rho_g**	3.12	2.59	3.78	3.00
**beta_Pl**	4.49	3.45	5.52	5.00

Table S1.3.4. Estimated vs given parameter values for the 'Uninformative' run

Mean and 95%CI refer to the posterior estimates, while given refers to the 'true' parameter values used to simulate the dataset. Priors for log_alpha_Vu and log_beta_Vu were set to ~U(-100,100) and ~U(1,5), respectively. All other priors are set as stated in the main text (Table 2).

	Mean	95%CI_low	95%CI_upp	given
**log_alpha_Vu**	-4.10	-4.28	-3.92	-4.04
**log_beta_Vu**	2.82	2.75	2.89	2.80
**sigma_rhoo_mu**	0.07	0.06	0.09	0.06
**sigma_rhoo_sigma**	0.06	0.04	0.08	0.04
**sigma_sigma_Vo**	0.01	0.00	0.03	0.01
**mu_sigma_Vo**	0.05	0.04	0.06	0.05
**sigma_sigma_Lo**	0.05	0.01	0.09	0.01
**mu_sigma_Lo**	0.21	0.18	0.24	0.20
**mu_rho_g**	1031.42	1030.70	1032.12	1031.00
**sigma_rho_g**	2.68	2.23	3.24	3.00
**beta_Pl**	4.76	3.66	5.89	5.00

Table S1.3.5. Estimated vs given global parameter values for the 'mis-specified' run

Mean and 95%CI refer to the posterior estimates, while given refers to the 'true' parameter values used to simulate the dataset. Priors are set as stated in the main text (Table 2).

	Mean	95%CI_low	95%CI_upp	given
**log_alpha_Vu**	-3.85	-3.96	-3.74	-4.5
**log_beta_Vu**	3.24	3.19	3.29	3.5
**sigma_rhoo_mu**	0.05	0.04	0.06	0.06
**sigma_rhoo_sigma**	0.04	0.03	0.05	0.04
**sigma_sigma_Vo**	0.00	0.00	0.01	0.01
**mu_sigma_Vo**	0.04	0.04	0.05	0.05
**sigma_sigma_Lo**	0.06	0.02	0.11	0.01
**mu_sigma_Lo**	0.22	0.19	0.27	0.2
**mu_rho_g**	1029.39	1028.52	1030.29	1030
**sigma_rho_g**	3.44	2.86	4.18	3
**beta_Pl**	6.41	5.24	7.62	5

Table S1.3.6. Estimated vs given global parameter values for the 'check1' run

Mean and 95%CI refer to the posterior estimates, while given refers to the 'true' parameter values used to simulate the dataset. Priors are set as stated in the main text (Table 2).

	Mean	95%CI_low	95%CI_upp	given
**sigma_rhoo_mu**	0.06	0.05	0.07	0.06
**sigma_rhoo_sigma**	0.04	0.03	0.06	0.04
**mu_rho_g**	1030.93	1030.29	1031.57	1031.00
**sigma_rho_g**	2.94	2.53	3.42	3.00
**beta_Pl**	4.92	4.34	5.49	5.00
**alpha_BC**	0.00	-0.02	0.01	0.00
**mu_sigma_BC**	0.05	0.05	0.06	0.05
**sigma_sigma_BC**	0.01	0.01	0.02	0.01

Table S1.3.7. Posterior parameter estimates for the check1 model fitted to observed data

Priors and likelihood are given in the main text (Table 2) and this supplement (Check1model), respectively.

	Mean	95%CI_low	95%CI_upp
**sigma_rhoo_mu**	0.07	0.06	0.08
**sigma_rhoo_sigma**	0.04	0.03	0.05
**mu_rho_g**	1029.03	1028.58	1029.5
**sigma_rho_g**	1.65	1.35	2.03
**beta_Pl**	7.62	5.3	10.14
**alpha_BC**	-0.04	-0.07	-0.01
**mu_sigma_BC**	0.1	0.07	0.16
**sigma_sigma_BC**	0.07	0.03	0.15

Table S1.3.8. Posterior parameter estimates from the full model fitted to data with large males removed

Priors and likelihood are given in the main text (Table 2) and this supplement (Full model), respectively.

	Mean	95%CI_low	95%CI_upp
**log_alpha_Vu**	-4.01	-4.13	-3.89
**log_beta_Vu**	2.89	2.83	2.94
**sigma_rhoo_mu**	0.07	0.06	0.08
**sigma_rhoo_sigma**	0.04	0.03	0.05
**sigma_sigma_Vo**	0.05	0	0.12
**mu_sigma_Vo**	0.1	0.07	0.16
**sigma_sigma_Lo**	0.15	0.02	0.39
**mu_sigma_Lo**	0.26	0.16	0.43
**mu_rho_g**	1029.05	1028.56	1029.54
**sigma_rho_g**	1.66	1.36	2.05
**beta_Pl**	6.01	1.51	10.5

S1.4. Supplementary tables

Table S1. Response to tagging and UAV

Response codes are as follows: 0 = No response; 1 Low response - Brief and mild, e.g. fast dive, change in speed or orientation; 2: Moderate Response – More forceful reaction but not prolonged, e.g. breach, tail slap; 3: Strong response – continued forceful reaction, multiple tail slaps/breaches/trumpet blows or sustained flight.

Date	Deployment ID	Tagger	Method	Response code (tagging)	UAV flown	UAV Pilot	Response code (UAV)
**08/07/2021**	sw21_189a	RP	Cantilever	1	y	AB	0
**15/07/2021**	sw21_196a	RP	Cantilever	1	y	AB	1
**23/07/2021**	sw21_204a	RP	Cantilever	1	y	AB	0
**28/07/2021**	sw21_209a	RP	Cantilever	1	n	-	-
**30/07/2021**	sw21_211a	RP	Cantilever	2	y	AB	0
**03/08/2021**	sw21_215a	RP	Cantilever	2	y	AB	0
**03/08/2021**	sw21_215b	RP	Cantilever	2	n		
**09/08/2021**	sw21_221a	RP	Cantilever	1	y	AB	0
**18/08/2021**	sw21_230a	AB	Hand pole	1	y	AB	0
**18/08/2021**	sw21_230b	AB	Hand pole	1	y	AB	0
**20/08/2021**	sw21_232a	AB	Hand pole	1	y	AB	0
**20/08/2021**	sw21_232b	AB	Hand pole	1	n	-	-
**30/08/2021**	sw21_242a	RP	Cantilever	1	y	AB	0
**07/09/2021**	sw21_250a	RP	Cantilever	2	y	AB	0
**07/09/2021**	sw21_250b	RP	Cantilever	2	n	-	-
**30/06/2022**	sw22_181a	AB	Cantilever	1	y	RP	0
**06/07/2022**	sw22_187a	AB	Cantilever	1	n	-	-
**07/07/2022**	sw22_188a	AB	Cantilever	1	n	-	-
**11/07/2022**	sw22_192a	AB	Cantilever	1	y	AB	0
**20/07/2022**	sw22_201a	AB	Cantilever	3	y	AB	0
**20/07/2022**	sw22_201b	AB	Cantilever	1	y	AB	0
**28/07/2022**	sw22_209a	AB	Cantilever	2	y	AB	0
**29/07/2022**	sw22_210a	AB	Cantilever	1	y	AB	0
**04/08/2022**	sw22_216a	AB	Cantilever	2	y	AB	0
**11/08/2022**	sw22_223a	AB	Cantilever	1	y	AB	0
**12/08/2022**	sw22_224a	AB	Cantilever	2	y	AB	0
**17/08/2022**	sw22_229a	AB	Cantilever	1	y	AB	0
**22/08/2022**	sw22_234a	AB	Cantilever	1	y	AB	0
**22/08/2022**	sw22_234b	AB	Cantilever	1	y	AB	0

Table S2. Allometric expressions used to estimate tissue compartment masses.

Multiplication of length by 3.281 converts the units of total body length from meters to feet where required, allowing coefficients to be conveniently compared to those reported in the papers cited. All outputs are in kg. The distribution parameters are mean and standard deviation for Gaussian distributions, and minimum and maximum for uniform distributions.

Compartment	Allometric expression	Details	Source(s)
**Skeleton**	$M_{[w,c=1]} = exp(-2.86 + log(L \cdot 3.281) \cdot 2.786 + \sim Gaussian(0, 0.098)$	Raw whaling data were reanalysed. A two parameter allometric function was fitted, with both parameters estimated from the data. Draws from a normal distribution, parameterised using the model residuals, were used to approximate log error.	(Omura, 1950)
**Muscle**	$M_{[w,c\,=\,2]} = exp(-0.949 + log(L \cdot 3.281) \cdot 2.579 + \sim Gaussian(0, 0.160)$	As for Skeleton, above.	(Ohno and Fujino, 1952; Omura, 1950)
**Organs**	$M_{[w,c=3]} = exp(4.254 + log(L \cdot 3.281) \cdot 0.680 + \sim Gaussian(0, 0.259)$	As for Skeleton, above.	(Omura, 1950)
**Viscera**	$M_{[w,c=4]} = exp(-3.408 + log(L \cdot 3.281) \cdot 2.700 + \sim Gaussian(0, 0.286)$	Includes visceral fat and unnamed organs. Modelled separately from organs to allow for different composition of visceral fat.	(Omura, 1950)

**Blubber**	$M_{[w,c=5]} = exp(-1.504 + log(L \cdot 3.281) \cdot 2.741 + \sim Gaussian(0, 0.166)$	As for Skeleton, above.	(Ohno and Fujino, 1952; Omura, 1950)
**Sound production apparatus (SPA)**	$M_{[w,c=6]} = exp(-4.402 + log(L \cdot 3.281) \cdot 3.100 + \sim Gaussian(0, 0.192)$	As for skeleton, but the model was fitted to sound production apparatus mass, calculated from spermaceti yield data from Omura and Clarke, assuming 98.6% lipid composition of the apparatus, as reported by Clarke.	(Clarke, 1978a; Ohno and Fujino, 1952; Omura, 1950)
**Other**	$M_{[w,c=7]} = exp(-8.094 + log(L \cdot 3.281) \cdot 4.140 + \sim Gaussian(0, 0.287)$	Includes the tongue, spermaceti case and other tendons and some 'scraps' of meat and bone not weighed separately. As for Skeleton, above.	(Omura, 1950)
**Blood**	$M_{[w,c = blood]} = \sum_{c=1}^{7} M_{[c]} \cdot \sim Gaussian(0.148, 0.0125)$	Strictly, this compartment is blood not retained in other tissues sampled, rather than total blood mass. Fluid loss is an unavoidable consequence of weighing bodies in pieces, but it is possible that differences in methodology between studies led to differences in blood lost. For this reason, and to avoid bias from 'double counting' we opted to sample from a relatively broad range of plausible proportionate blood masses, relative to uncorrected predicted allometric mass but centred the distribution on a	(Lockyer, 1991; Omura, 1950; Rice, 1989; Sleet et al., 1981)

	value obtained from a single adult female whale weighed first whole and then in pieces.

Table S3. Parameterisation of tissue compartment compositions used in Monte Carlo simulations.

The distribution parameters are mean and standard deviation and minimum and maximum for Gaussian and uniform distributions, respectively.

Compartment	Proportion by mass of protein (p_p)	Proportion by mass of water (p_w)	Proportion by mass of mineral ash (p_a)	Proportion by mass of lipid (p_l)	Details	Source (s)
**Skeleton**	$\sim Gaussian(0.22, 10^{-5}, truncated\ at\ 0,1)$	$\sim Gaussian(0.25, 10^{-5}, truncated\ at\ 0,1)$	$\sim Gaussian(0.25, 10^{-5}, truncated\ at\ 0,1)$	$\sim Gaussian(0.26, 10^{-5}, truncated\ at\ 0,1)$	Mean of all measurements of skeletal tissue composition (excluding cartilage), weighted by the contribution of the type of bone measured to total skeleton mass, with a small amount of variability added.	(Omura, 1950; Watanabe and Suzuki, 1950)
**Muscle**	$\sim Gaussian(0.26, 0.0086, truncated\ at\ 0,1)$	$\sim Gaussian(0.72, 0.0082, truncated\ at\ 0,1)$	$\sim Gaussian(0.001, 0.0004, truncated\ at\ 0,1)$	$\sim Gaussian(0.03, 0.001, truncated\ at\ 0,1)$	Samples are taken across the whole body, but it is not known how much each region contributes to the total. No raw data or ranges were reported by Lockyer, but plots and text suggest a unimodal distribution. We therefore opted to draw from a normal distribution using the mean and SD across all sampling locations.	(Lockyer, 1991)
**Organs**	$\sim Unif(0.167, 0.25)$	$\sim Unif(0.7, 0.77)$	$\sim Unif(0.0092, 0.0123)$	$\sim Unif(0.023, 0.044)$	Raw data from Lockyer. Since the relative masses of the organs were not reported and compositions of all organs was similar, uniform distributions from minimum and maximum values across all reported were used.	(Lockyer, 1991)
**Viscera**	$\sim Unif(0.016, 0.21)$	$\sim Unif(0.07, 0.77)$	$\sim Unif(0.00002, 0.012)$	$\sim Unif(0.024, 0.80)$	This includes non-named organs and visceral fat. We therefore used a uniform distribution covering the full range of	(Lockyer, 1991;

					composition values reported for both tissues.	Omura, 1950)
**Blubber**	$\sim Unif(0.09, 0.35)$	$\sim Unif(0.25, 0.45$	$\sim Unif(0.002, 0.005)$	$\sim Gaussian(0.49, 0.179, truncated\ at\ 0,1)$	Little is known about how of lipid and water covary in sperm whales, but both are highly variable. We also wanted to avoid fatty tissues strongly influencing the lean mass and density estimates. Independent distributions covering the full range of empirical observations in the literature were therefore used.	(Evans et al., 2003; Lockyer, 1991)
**Sound production apparatus**	$\sim Unif(0, 0.01)$	$\sim Unif(0, 0.01)$	$\sim Unif(0, 0.0001)$	$\sim Unif(0.98, 0.99)$	Clarke reported 98.8-and 98.4% lipids by mass for the spermaceti and junk respectively. We therefore assumed 98-99% lipid by mass, negligible ash, and 0-1% for the remaining components.	(Clarke, 1978a; Omura, 1950)
**Other**	$\sim Gaussian(0.26, 0.0086, truncated\ at\ 0,1)$	$\sim Gaussian(0.72, 0.0082, truncated\ at\ 0,1)$	$\sim Unif(0, 0.05)$	$\sim Gaussian(0.03, 0.001, truncated\ at\ 0,1)$	This is likely to be mostly muscle and connective tissue but may include some small bones. We therefore used a uniform distribution based on the values for muscle, adjusted to allow for a larger proportion of mineral ash.	(Lockyer, 1991)
**Blood**	0.193	0.8	0.005	0.002	Only one direct measurement was available, and composition is unlikely to vary significantly: the properties of blood being subject to tight physiological control. We therefore opted to use a single point value for this term.	(Watanabe and Suzuki, 1950)

Table S4. Posterior global parameter estimates from Bayesian model fitting

See Appendix S1.3 and Table 1 in the main text for definition of parameters.

Name in text	Name in NIMBLE code	Mean	95%CI_low	95%CI_upp
$\log(\alpha_v)$	log_alpha_Vu	-4	-4.12	-3.88
$\log(\beta_v)$	log_beta_Vu	2.88	2.83	2.93
$\overline{\sigma_{\rho o}}$	mu_sigma_rhoo	0.07	0.06	0.08
$\sigma_{\sigma\rho o}$	sigma_sigma_rhoo	0.04	0.03	0.05
$\sigma_{\sigma Vo}$	sigma_sigma_Vo	0.04	0.01	0.1
$\overline{\sigma_{Vo}}$	mu_sigma_Vo	0.09	0.06	0.14
$\sigma_{\sigma Lo}$	sigma_sigma_Lo	0.17	0.02	0.43
$\overline{\sigma_{Lo}}$	mu_sigma_Lo	0.29	0.19	0.47
$\overline{\rho_u}$	mu_rho_g	1029.07	1028.6	1029.54
$\sigma_{\rho u}$	sigma_rho_g	1.63	1.33	2.02
$\beta_{\delta P_l}$	beta_Pl	6.75	2.63	10.96

S1.5. Supplemental figures

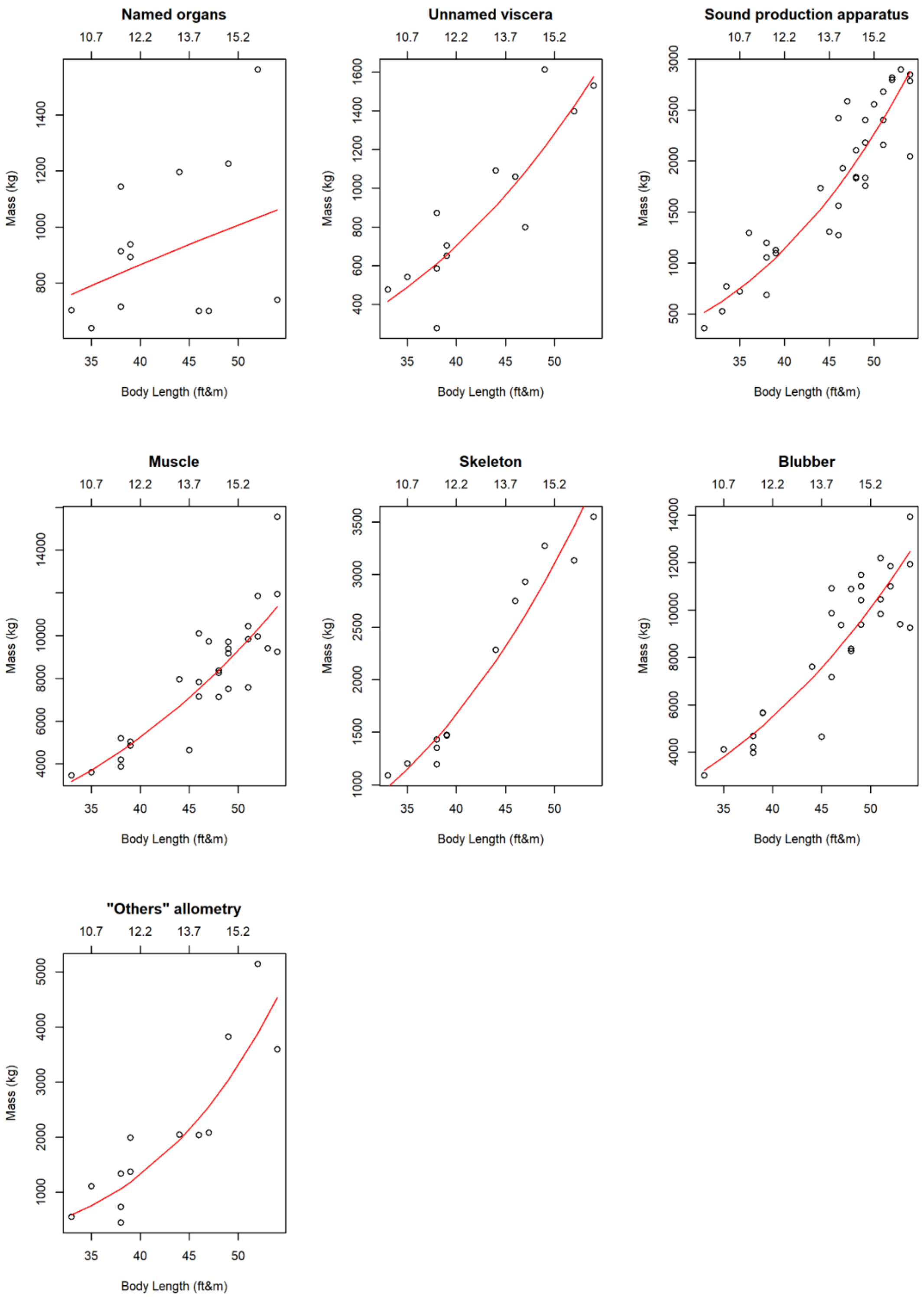

Figure S1. Allometric models of tissue compartment masses

Points show the raw data from the sources described in Table S2. X axis labels show length in feet (bottom) and meters (top)

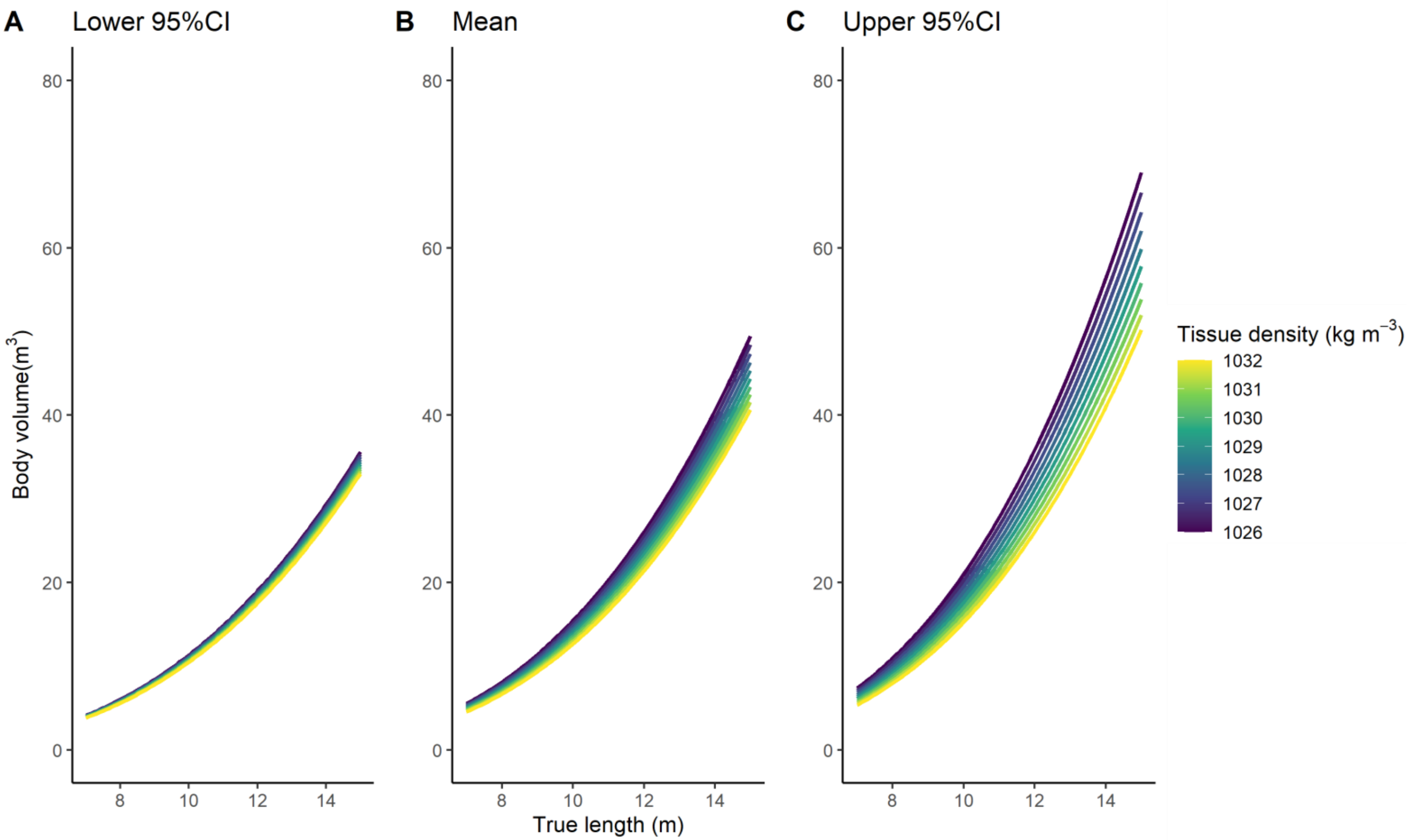

Figure S2. Model predictions for sperm whale body volume as a function of length and tissue density. *Predictions are from A the lower 95% posterior CIs for model coefficients, B: posterior mean coefficients and C: upper 95% posterior CIs.*

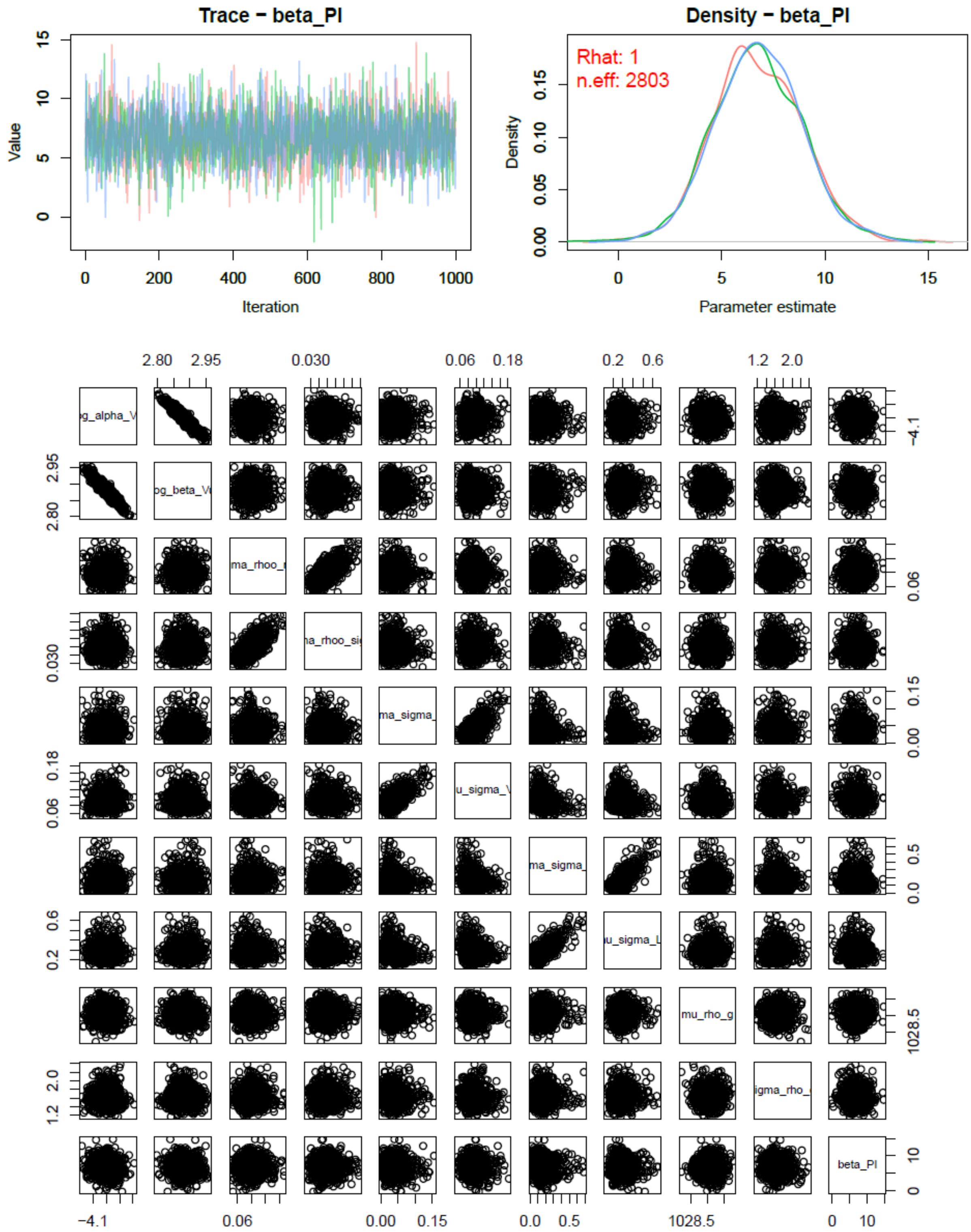

Figure S3. Example trace (top left) density (top right) and identifiability matrix (bottom) diagnostic plots generated from the posterior samples from the final model fit

Full diagnostics are too large to reproduce here but are available in the digital supplementary information (under Outputs/MCMC_diagnostics).

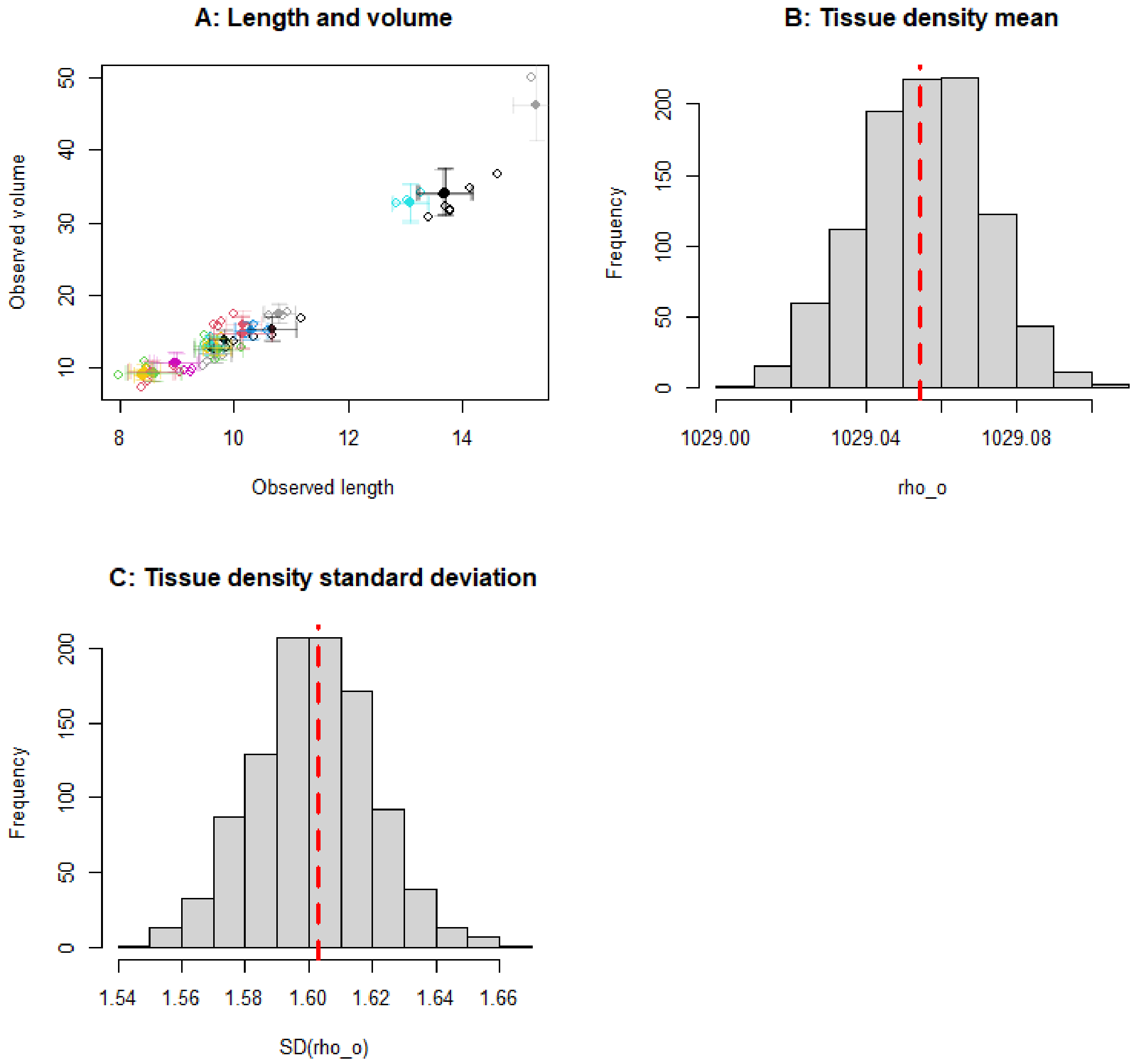

Figure S4. Posterior predictive checks

Panel A shows posterior predictive mean and SD (Solid points and error bars) length and volume vs the raw observed data (open points) for each whale (colour). Panels B and C show posterior predictions of mean and standard deviation of observed tissue density, respectively (Histograms) overlaid with those calculated directly from the raw observed data (red dashed lines).

References

Aoki, K., Isojunno, S., Bellot, C., Iwata, T., Kershaw, J., Akiyama, Y., Martín López, L.M., Ramp, C., Biuw, M., Swift, R.R., Wensveen, P.J., Pomeroy, P., Narazaki, T., Hall, A.J., Sato, K., Miller, P.J.O., 2021. Aerial photogrammetry and tag-derived tissue density reveal patterns of lipid-store body condition of humpback whales on their feeding grounds. Proc. R. Soc. 288, 20202307. https://doi.org/10.1098/rspb.2020.2307

Bierlich, K., Schick, R., Hewitt, J., Dale, J., Goldbogen, J., Friedlaender, A., Johnston, D., 2021. Bayesian approach for predicting photogrammetric uncertainty in morphometric measurements derived from drones. Mar. Ecol. Prog. Ser. 673, 193–210. https://doi.org/10.3354/meps13814

Burnett, J.D., Lemos, L., Barlow, D., Wing, M.G., Chandler, T., Torres, L.G., 2018. Estimating morphometric attributes of baleen whales with photogrammetry from small UASs: A case study with blue and gray whales. Mar. Mammal Sci. https://doi.org/10.1111/mms.12527

Christiansen, F., Vivier, F., Charlton, C., Ward, R., Amerson, A., Burnell, S., Bejder, L., 2018. Maternal body size and condition determine calf growth rates in southern right whales. Mar. Ecol. Prog. Ser. 592, 267–281. https://doi.org/10.3354/meps12522

Clarke, M.R., 1978a. Structure and Proportions of the Spermaceti Organ in the Sperm Whale. J. Mar. Biol. Assoc. U. K. 58, 1–17. https://doi.org/10.1017/S002531540002

Clarke, M.R., 1978b. Physical properties of spermaceti oil in the sperm whale. J. Mar. Biol. Assoc. U. K. 58, 19–26. https://doi.org/10.1017/S0025315400024383

DJI, 2020. DJI Pilot [WWW Document]. URL https://www.dji.com/uk/downloads/djiapp/dji-pilot (accessed 10.31.22).

Ellis, K.J., 2000. Human Body Composition: In Vivo Methods. Physiol. Rev. 80, 649–680. https://doi.org/10.1152/physrev.2000.80.2.649

Evans, K., Hindell, M.A., Thiele, D., 2003. Body fat and condition in sperm whales, Physeter macrocephalus, from southern Australian waters. Comp. Biochem. Physiol. - Mol. Integr. Physiol. 134, 847–862. https://doi.org/10.1016/S1095-6433(03)00045-X

Glarou, M., Gero, S., Frantzis, A., Brotons, J.M., Vivier, F., Alexiadou, P., Cerdà, M., Pirotta, E., Christiansen, F., 2023. Estimating body mass of sperm whales from aerial photographs. Mar. Mammal Sci. 39, 251–273. https://doi.org/10.1111/mms.12982

Kelp Marine, n.d. Kelp Marine Research [WWW Document]. URL https://kelpmarineresearch.com/index.php?page=about (accessed 6.21.24).

Keys, A., BroŽek, J., 1953. Body Fat in Adult Man. Physiol. Rev. 33, 245–325. https://doi.org/10.1152/physrev.1953.33.3.245

LightWare Optoelectronics, 2018. SF11-Laser-Altimeter-Manual [WWW Document]. URL https://www.mouser.com/datasheet/2/321/28054-SF11-Laser-Altimeter-Manual-Rev8-1371857.pdf

Lockyer, C., 1991. Body composition of the sperm whale, Physeter catodon , with special reference to the possible functions of fat depots. J. Mar. Res. Inst. Reyk. 12, 1–25.

Lonati, G.L., Singleton, E.M., Phelps, C.E., Koopman, H.N., Pabst, D.A., 2019. The density of odontocete integument depends on blubber lipid composition and temperature. Mar. Mammal Sci. 35, 595–616. https://doi.org/10.1111/mms.12554

Miller, P.J.O., Narazaki, T., Isojunno, S., Aoki, K., Smout, S., Sato, K., 2016. Correction: Body density and diving gas volume of the northern bottlenose whale (Hyperoodon ampullatus). J. Exp. Biol. 219, 2962–2962. https://doi.org/10.1242/jeb.148841

Moore, F.D., Olsen, K.H., McMurray, J.D., Parker, H.V., Ball, M.R., Boyden, C.M., 1963. The Body Cell Mass and its Supporting Environment: Body Composition in Health and Disease. W. B. Saunders, Philadelphia.

Ohno, M., Fujino, K., 1952. Biological Investigation on the Whales Caught by the Japanese Antarctic Whaling Fleets, season 1950/51. Sci. Rep. Whales Res. Inst. 7, 125-190.
Omura, H., 1950. On the Body. Weight of Sperm and Sei Whales located in the Adjacent Waters of Japan. Sci. Rep. Whales Res. Inst. 4, 1–13.
Rice, D.W., 1989. Sperm whale Physeter macrocephalus Linnaeus, 1758. Handb. Mar. Mamm. 4, 177–233.
Siri, W.E., 1956. Body composition from fluid spaces and density: analysis of methods. Nutr. Burbank Los Angel. Cty. Calif 9, 480–491.
Sleet, R.B., Sumich, J.L., Weber, L.J., 1981. Estimates of total blood volume and total body weight of a sperm whale (*Physeter catodon*). Can. J. Zool. 59, 567–570. https://doi.org/10.1139/z81-083
Watanabe, H., Suzuki, K., 1950. Chemical Compcsition of Various Parts of Sperm Whaie. 日本水産学会誌 15, 735–740. https://doi.org/10.2331/suisan.15.735
